# Health Insurance Coverage Changes Under the Affordable Care Act Among High Housing Cost Households, 2010–18

**DOI:** 10.1002/hec.4912

**Published:** 2024-11-13

**Authors:** Yu Cao, Yuxin Su, Guan Wang, Chengcheng Zhang

**Affiliations:** ^1^ EAPCE Research Center World Bank Group Washington DC USA; ^2^ SKEMA Centre for Sustainability Studies SKEMA Business School Suzhou China; ^3^ Brown School of Business and Leadership Stevenson University Owings Mills Maryland USA; ^4^ Department of Health Sciences Towson University Towson Maryland USA

**Keywords:** Affordable Care Act, health insurance coverage, housing affordability, low‐income households, Medicaid expansion

## Abstract

This study examines the impact of the Affordable Care Act (ACA) on health insurance coverage among rent‐burdened households—those spending more than 30% of their income on rent—and non‐rent‐burdened households. Using data from American Community Survey, we find that Medicaid take‐up rate increased 8.88 percentage points (pp) among rent‐burdened households and 7.54 pp among non‐rent‐burdened households in expansion states. Conditional on household income and demographic characteristics, rent‐burdened households exhibit a 1.5 pp higher likelihood of Medicaid enrollment, with an additional decline of 0.7 pp in employer‐sponsored insurance and 1.0 pp in directly purchased insurance enrollment. These effects were more pronounced among individuals aged over 26 and those in states without state‐run exchanges. The findings show the importance of tailored Medicaid policies to assist households facing housing burdens, especially for those ineligible for housing vouchers.

## 1 Introduction

Housing, a critical social determinant of health, affects well‐being through affordability, stability, quality, and safety (Taylor 2018; Gibson et al. 2011). For low‐income renters, the high and inflexible cost of rent consumes a large portion of the household budget, creating financial constraints that limit spending on other essentials such as healthcare (Hess et al. 2024; Fernald 2020; Kirkpatrick and Tarasuk 2011). The 2008 Great Recession drove up rent burdens and heightened financial stress. Increased foreclosures force many households into renting, contributing to a rise in rent‐to‐income ratios (Colburn and Allen 2018). In 2019, nearly half of renter households in the United States were rent‐burdened, spending more than 30% of their income on rent; about a quarter were severely rent‐burdened, spending over 50% (Fernald 2020; Hess et al. 2024).^1^ Goodman and Ganesh (2017) found that 92 percent of rent‐burdened renters are from the low‐income group. High rental costs often lead to residential instability, longer commutes, and less time for health‐promoting activities such as exercise and sleep (Kirkpatrick and Tarasuk 2011; Swope and Hernández 2019). In addition, individuals facing financial pressures may be forced to make difficult trade‐offs between housing and essentials such as fresh food and health insurance, thereby increasing their exposure to health risks (Mehdipanah 2023; Gibson et al. 2011; Kaplan and Porter 2011; Baker et al. 2020).

Low‐to medium‐income individuals, who are more likely to face financial constraints, often have greater healthcare needs. Access to affordable health insurance is essential to alleviate these financial burdens and reduce exposure to health risks (Glied, Collins, and Lin 2020; Hu et al. 2018; Simon, Soni, and Cawley 2017). The Affordable Care Act (ACA) was designed to improve healthcare access for vulnerable populations by expanding Medicaid and providing financial assistance through the ACA Marketplace. Previous studies have shown that the ACA Medicaid expansion significantly increased Medicaid coverage and reduced uninsured rates, particularly in expansion states (Sommers et al. 2015; Courtemanche, Marton, and Yelowitz 2016; Courtemanche et al. 2017, 2019; Wherry and Miller 2016; Frean, Gruber, and Sommers 2016, 2017; Kaestner et al. 2017; Kominski, Nonzee, and Sorensen 2017, Kominski et al. 2020a, 2020b; Duggan, Goda, and Jackson 2019; Chu 2023).

In addition to increasing insurance coverage, the ACA has significantly extended healthcare access for vulnerable groups, including racial minorities such as Hispanics and non‐Hispanic Blacks (Wehby and Lyu 2018; Soni, Hendryx, and Simon 2017; Ye and Rodriguez 2021), adults with disabilities (Creedon et al. 2022), and workers in sectors such as service, farming, construction, and transportation (Agarwal, Goldman, and Sommers 2019). However, the impact of ACA on households with high rent burdens remains underexplored. Research indicates that high rent‐burdened households, mostly low‐to medium‐income renters, face higher risks of eviction, financial distress, and unmet healthcare needs (Shamsuddin and Campbell 2022). Therefore, it is important to investigate not only whether Medicaid expansion benefited high rent‐burdened households, but also whether the extent of these benefits differed across households with varying levels of housing affordability.

Our paper addresses this gap by examining how health insurance coverage has changed since the ACA's implementation among low‐to medium‐income households, particularly those with varying degrees of rent burden. We hypothesize that households in rent‐burdened groups in Medicaid expansion states will exhibit greater increases in Medicaid enrollment than those in non‐expansion states. Given the heightened risk of unforeseen healthcare expenses, we further expect that rent‐burdened households are more likely to benefit more from Medicaid expansion than their non‐rent‐burdened counterparts.

Using data from the American Community Survey (ACS), we employ a difference‐in‐difference (DID) approach to estimate and compare the pre‐post coverage changes between expansion and non‐expansion states, as well as among households with divergent levels of housing affordability. We treat 2014 as a transition year and examine health insurance coverage trends between 2010–2013 and 2015–2018 for individuals in expansion and non‐expansion states, separating the analysis into rent‐burdened and non‐rent‐burdened categories. Additionally, we leverage the difference‐in‐difference‐in‐difference (DDD) approach to determine and compare the average treatment effect of Medicaid expansion between these distinct housing affordability groups. To ensure the robustness of our results, we conduct several robustness checks, which confirm the validity of our findings. Finally, we supplement our investigation with an event study, tracking the trajectory of health insurance coverage after the ACA expansion.

The estimation results indicate that the ACA increased overall health insurance coverage, regardless of the level of housing affordability, in both expansion and non‐expansion states, which is consistent with previous studies (Courtemanche et al. 2017; Sommers et al. 2015; Kaestner et al. 2017; Frean, Gruber, and Sommers 2016). For example, after the ACA was implemented, the uninsured rate decreased by 13.35 and 10.20 percentage points (pp) in expansion and non‐expansion states, respectively. The adjusted Medicaid coverage increased by an additional 8.08 pp in expansion states compared to non‐expansion states for those with annual income up to 400% of the Federal Poverty Level (FPL). Among the rent‐burdened group, Medicaid coverage improved by 8.88 pp more in expansion states than non‐expansion states. Furthermore, within expansion states, the pre‐post Medicaid coverage change for the rent‐burdened group exceeded that observed for the non‐rent‐burdened group by 1.5 pp. By contrast, the directly‐purchased insurance coverage ratio experienced an additional 1.0 pp decrease in the rent‐burdened group after the expansion of the ACA went into effect.

Our paper builds on the growing literature covering the effects of the ACA on health insurance coverage, particularly for vulnerable populations such as low‐income individuals (Sommers et al. 2017b; Wehby and Lyu 2018; Ye and Rodriguez 2021; Creedon et al. 2022; Hamilton 2024). However, not enough research has explored the varying effects the changes in ACA's coverage have had on households with different rent burdens. While some low‐income households benefit from housing assistance programs that help alleviate financial constraints, many individuals with incomes above the eligibility thresholds still experience significant financial burdens. The ACA's Medicaid expansion, however, provides critical support for these individuals by extending health insurance coverage to those who are not eligible for affordable housing programs but continue to face financial challenges. Our results show that Medicaid expansion provides a safety net for individuals who may not qualify for housing assistance but still have limited resources. In particular, our triple‐difference estimates suggest that individuals in rent‐burdened household benefit more from the ACA's implementation.

While our study focuses on rent burden, it is important to acknowledge that other household expenses, such as debt and childcare costs, also significantly impact household budgets. Rent was chosen as our focus because of its unique characteristics: it is typically an inflexible cost that cannot be easily and quickly adjusted without incurring additional upfront costs, making it a distinct financial burden. Unfortunately, the American Community Survey does not include comprehensive data on other financial constraints like debt and childcare costs. This limitation precludes us from analyzing these factors directly. However, we approximate these costs by adding additional controls in our analysis. For instance, we included the number of children under 16 as a proxy for childcare‐related expenses and incorporated county‐level fixed effects to partially control for variations in living costs. As a robustness check, we also included variables such as the total number of children and household members, as well as household costs like electricity, fuel, gas, and water expenses. These additional controls yielded results that were consistent with our benchmark findings. It is important to emphasize again that we chose rent as the primary focus due to its inflexibility and the disproportionate financial pressure it places on households. Unlike other household expenses, rent is a fixed cost that cannot be easily adjusted, making it a critical factor in understanding the financial constraints faced by low‐to medium‐income families.

This paper proceeds as follows. Section 2 provides the institutional background of ACA Medicaid expansion and reviews the literature on housing. Section 3 explains the empirical methods used in the analysis. Section 3.1, Section 3.2, and Section 3.3 describe the data source, target population, and empirical strategy, respectively. The results are presented in Section 4, followed by a discussion and conclusion in Section 5.

## 2 ACA Expansion, Housing Affordability and Health Outcome

Prior to ACA expansion, each state determined its own eligibility for coverage, which included Medicaid for individuals with incomes up to 100% of the federal poverty limit. Because it was a state‐by‐state decision, some vulnerable populations were excluded, especially in states that had tight requirements for Medicaid eligibility. As of January 1, 2014, 29 states that adopted the ACA expansion extended Medicaid eligibility to individuals with incomes up to 138% of the FPL.^2^ Additionally, ACA created state and federal health insurance exchanges for individuals with incomes between 138% and 400% FPL, providing premium tax credits and cost‐sharing reductions to make private health insurance more affordable for those not eligible for Medicaid but cannot afford the full cost of regular health insurance. The ACA expansion is particularly crucial for addressing the financial challenges faced by low‐to medium‐income households. Housing affordability, as a major cost in their family expenses, is not just an economic issue, but also a significant influencing factor to their health outcomes.

Health outcomes for low‐to medium‐income renters, who are more likely to experience rent burden, can be significantly affected in three ways. First, high housing costs may limit financial resources available for other essential expenses. Households with constrained budgets often face tough trade‐offs between covering housing‐related costs and investing in health‐promoting activities or healthcare services. Previous studies have found a positive association between high housing costs and reduced medical expenses such as routine doctor visits, health care, or prescribed medicines (Meltzer and Schwartz 2016; Pollack, Griffin, and Lynch 2010), increasing exposure to health risks (Mehdipanah 2023; Colburn et al. 2024). Second, these low‐income households frequently reside in substandard housing, exposing them to environmental hazards that can directly impair their health (Evans 2021; Arcaya, Ellen, and Steil 2024). Prior research has shown that substandard housing conditions, such as overcrowding, inadequate heating or cooling, poor air and water quality, can exacerbate health problems like asthma and other respiratory disease (Krieger and Higgins 2002; Ahmad et al. 2020; Swope and Hernández 2019). Third, severe rent burden can lead to increased housing instability, with a higher likelihood of evictions (Ramphal et al. 2023; Desmond 2018; Smith et al. 2024; Hoke and Boen 2021). Such instability often results in frequent relocations, which can disrupt medical care continuity and escalate psychological stress, thereby worsening chronic health conditions (Garcia, Doran, and Kushel 2024; Decker et al. 2024; Ramphal et al. 2023).

The ACA expansion has significantly increased health insurance coverage among populations in rent burdened household—defined as households spending in excess of 30% of their income on housing. Data from the ACS indicate that households with incomes below 100% of the FPL allocate approximately 49.8% of their earnings to rent. Among these households, 75% are considered rent burdened, and 45.4% are severely rent burdened, spending more than 50% of their income on rent. For households earning between 100% and 138% FPL, the average rent expenditure is 42.5% of their income, with 70% classified as rent burdened and 30.1% as severely rent burdened.

By raising Medicaid eligibility threshold to include individuals with incomes up to 138% FPL, the ACA has ensured that a larger fraction of vulnerable households, previously uncovered, now have access to Medicaid. This expansion not only facilitates access to essential healthcare services but also alleviates some of the financial pressures these households face, potentially improving their overall health outcomes. Moreover, for low‐to medium‐income households earning between 138% and 400% FPL, where 34% are rent burdened, the ACA's provisions for tax credits and cost‐sharing reductions further decrease the burden of healthcare costs. These financial aids enhance the affordability of health services and reduce the likelihood that these families will delay seeking medical care or purchasing health insurance, thus improving their financial and physical well‐being.

## 3 Method

### 3.1 Data Source

We estimate the impact of ACA Medicaid expansion on health insurance coverage among households with varying degrees of housing affordability. States are categorized based on their Medicaid expansion status and timing into early expansion states, 2014 expansion states, late expansion states, and non‐expansion states (see Supporting Information S1: Table C1). Our analysis utilizes data from the 2010–2018 waves of the ACS, a comprehensive cross‐sectional survey collected by the U.S. Census Bureau. The ACS covers more than 1% of the U.S. population and provides extensive data on health insurance, demographics, and socioeconomic variables including education, housing expenses, and household income. Compared to other household surveys, such as the CPS, the ACS offers more granular information on household housing expenditures and conditions, making it well‐suited for studying the intersection of both housing affordability and healthcare access.

By starting in 2010, we were able to collect baseline data before the full implementation of major ACA provisions. This approach also allowed us to establish the pre‐expansion trends in health insurance coverage. The identification of Medicaid expansion states and the timing of expansion are based on data from the Kaiser Family Foundation, which tracks state‐level policy changes related to Medicaid. This ensures precise alignment of state policy changes with the survey data.

We calculate the Federal Poverty Level using annual income cutoffs provided by the U.S. Department of Health and Human Services for each year from 2010 to 2018, adjusting for family size. While the ACS does not directly provide FPL data, it includes sufficient information on household income and family size, allowing us to determine FPL status.

### 3.2 Target Population

Our sample is restricted to U.S. citizens aged 16–64, with household incomes up to 400% of the FPL. We define housing affordability using the rent‐to‐income (RTI) ratio, classifying households with a RTI ratio up to 30% as non‐rent‐burdened and those above 30% as rent‐burdened. The 30% threshold has been the conventional standard for housing rental affordability since 1981. This benchmark was first introduced in the National Housing Act of 1937, which laid the foundation for the public housing program aimed at assisting low‐income families (Linneman and Megbolugbe 1992). Households that are not renting, or have a RTI ratio exceeding 100%, were excluded from our study. The cleaned sample comprises 1,055,772 individuals categorized as non‐rent‐burdened and 913,327 as rent‐burdened.

Our benchmark analysis focuses on individuals aged 18–64 to capture a comprehensive view of the working‐age population. One of the key provisions of the ACA allows young adults to remain on their parents' insurance until age 26. To examine the specific impacts of this provision, we conduct a stratified analysis by dividing the sample into two age groups: 18–26 and 27–64 (Supporting Information S1: Table E1). This approach enables us to explore the differential effects of Medicaid expansion and other ACA policies across these distinct age cohorts.

### 3.3 Empirical Strategy

The primary outcome variable is defined as a binary variable that equals one if a respondent is enrolled in Medicaid in a given year. We use a DID analysis to evaluate the impact of Medicaid expansion on Medicaid coverage rates across different RTI groups. Additionally, we extend our analysis to include uninsured rates and other health insurance types, such as directly‐purchased and employer‐sponsored private insurance.

In our benchmark analysis, we first estimate the impact of Medicaid expansion, which was implemented in 2014, on different health insurance coverage, regardless of household RTI ratio. We exclude states that adopted Medicaid expansion after 2014 but before 2018. Therefore, the benchmark analysis includes 37 states; 23 of which implemented Medicaid expansion in 2014. We estimate:

(1) $$Y_{\mathrm{ist}}^{f}=\beta \;Expanded_{s}\times Post_{t}+\psi_{1}Expanded_{s}+\psi_{2}Post_{t}+\gamma^{\prime }X_{\mathrm{ist}}+\eta_{s}+\eta_{t}+\varepsilon_{\mathrm{ist}}$$

where $Y_{\mathrm{ist}}$ is a dummy variable equals 1 if a household is enrolled in type f insurance. Here, we consider *Medicaid*, *employer‐sponsored*, *directly‐purchased*, and *uninsured*. $Expanded_{s}$ is a state‐level dummy variable that equals 1 if the household lives in a state that implemented Medicaid expansion in 2014. $Post_{t}$ is a year dummy variable that takes the value of 1 if the year is greater than 2014. $X_{i}$ is a vector of covariates that might affect a household's insurance enrollment decision. We include the household income, age, sex, education level, race, employment status, citizenship, and marital status. $\eta_{s}$ is the state fixed effect, and $\eta_{t}$ is the calendar year fixed effect. β represents the estimated average changes in type f health insurance coverage rates in expanded states relative to non‐expanded states.

We employ robust standard errors clustered at the PUMA (Public Use Microdata Areas) level to account for the potential correlation of the residual within each geographic unit. Clustering at the PUMA level is appropriate for our analysis because PUMA regions capture localized socioeconomic characteristics, which partially control for unobserved living costs that could potentially affect household budget constraints.^3^ PUMA boundaries, defined by the Census, were changed in 2010. The new PUMA code was adopted by ACS in 2012. To make geographic units comparable among these annual surveys from 2008 to 2018, we use ConsPUMAs created by Schroeder and Van Riper (2016). While PUMA‐level clustering captures localized effects that are possibly masked by state‐level aggregation, we also provide a robustness check with state‐level clustering in Section 4.5, confirming that our results remain consistent under this alternative specification.

To evaluate the ACA implementation on health insurance coverage across different RTI groups, we use an interacted DDD model. We estimate:

(2) $$\begin{array}{cc} Y_{\mathrm{ist}}^{f}= & \tilde{\beta }HighRent_{\mathrm{ist}}\times Expanded_{s}\times Post_{t}+\psi_{1}Post_{t}+\psi_{2}Expanded_{s}+\psi_{3}HighRent_{\mathrm{ist}}\\ & +\psi_{4}HighRent_{\mathrm{ist}}\times Expanded_{s}+\psi_{5}Highrent_{\mathrm{ist}}\times Post_{t}+\psi_{6}Expanded_{s}\times {Post}_{t}\\ & +\gamma^{\prime }X_{\mathrm{ist}}+\eta_{s}+\eta_{t}+\varepsilon_{\mathrm{ist}} \end{array}$$

$HighRent_{\mathrm{ist}}$ is a dummy variable which equals 1 if the household's annual rent payment is above 30% of annual income. Other controls are the same as Equation (1). $\psi_{6}$ measures the average changes in coverage rate after Medicaid expansion. $\tilde{\beta }$ is our triple difference estimator for the treatment effect on the high RTI group. It captures additional changes in coverage rate among the high RTI group households after Medicaid expansion. We use estimated $\tilde{\beta }$ to calculate the average treatment effect of Medicaid expansion on different RTI groups.

In addition to DID and DDD analysis, we also conduct the event study further to estimate the evolution of health insurance coverage after Medicaid expansion in 2014.^4^ We replace the dummy variable $Post_{t}$ with a set of year dummies and estimate:

(3) $$\begin{array}{cc} Y_{\mathrm{ist}}^{f}= & Expanded_{s}\times \sum_{y=2010}^{y=2012}\phi_{y}I\left(t=y\right)+Expanded_{s}\times \sum_{y=2014}^{y=2018}\beta_{y}\times I\left(t=y\right)\\ & +\psi_{1}Expanded_{s}+\gamma^{\prime }X_{\mathrm{ist}}+\eta_{s}+\eta_{t}+\varepsilon_{\mathrm{ist}} \end{array}$$

Here, I is an indicator variable which equals 1 if time equals year y. $\phi_{y}$ with y∈[2010,2012] is the estimated pre‐trend of the difference in insurance coverage f between expansion and non‐expansion states. $\beta_{y}$ with y∈[2014,2018] is the estimated average treatment effect on insurance coverage f in expansion states. Similarly, we replace $Post_{t}$ in Equation (2) with a sequence of indicator variables I to estimate the evolution of the average treatment effect of Medicaid expansion on different RTI groups.

## 4 Results

### 4.1 Descriptive Statistics

Summary statistics in Table 1 reveal significant disparities between high and non‐rent‐burdened groups in the full sample (columns (1) and (2)), as well as prior to Medicaid expansion (columns (3) and column (4)). The rent‐burdened group exhibits significant disadvantages compared to the non‐rent‐burdened group. For households with a rent‐to‐income ratio above 30%, 38.94% are in the 0%–100% FPL, and 61.06% are in the 101%–400% FPL. The unemployment rate and the percentage of female respondents are higher in the above 30% rent‐to‐income ratio group. This finding indicates the vulnerability of rent‐burdened households. Notably, even before the ACA's Medicaid expansion, people in the rent‐burdened households was more likely to be covered under Medicaid, with a coverage ratio of 29.72%, compared to 16.28% in the non‐rent‐burdened households. This finding shows that Medicaid has served as an important source of health insurance coverage for households facing financial burden prior to the Medicaid expansion. In the full sample, Medicaid coverage is significantly higher (32.60%) in the above 30% rent‐to‐income ratio group compared to the up to 30% group (18.53%). Employer‐sponsored insurance is more common (53.24%) in the non‐rent‐burdened households compared to the rent‐burdened households (32.95%). Directly‐purchased insurance and uninsured rates are higher in the rent‐burdened households. Overall, housing affordability interacts with income and other socio‐economic factors to shape insurance coverage outcomes.

**TABLE 1:** Descriptive statistics.

	Full sample		Prior‐ACA expansion	
	Up to 30%	Above 30%	Up to 30%	Above 30%
	(1)	(2)	(3)	(4)
Household income				
Income	55,298	26,803	50,965	24,871
0%–100% FPL	11.63%	38.94%	12.05%	41.08%
101%–400% FPL	88.37%	61.06%	87.95%	58.92%
Age	36.61	37.56	36.67	37.37
Unemployment rate	6.11%	9.19%	7.89%	11.85%
% Married household	33.84%	27.21%	34.73%	27.45%
% Female respondent	52.41%	57.18%	52.44%	57.30%
Race				
White	70.83%	65.22%	70.94%	65.21%
Black or African native	19.24%	24.30%	19.56%	24.99%
Asian	1.82%	2.22%	1.74%	1.97%
Multi‐racial	2.79%	3.14%	2.53%	2.90%
Other	5.32%	5.12%	5.23%	4.90%
Insurance coverage				
Medicaid	18.53%	32.60%	16.28%	29.72%
Employer‐sponsor	53.24%	32.95%	51.42%	30.61%
Direct‐purchase	8.00%	9.20%	6.42%	7.21%
Uninsured	20.58%	24.11%	25.85%	30.95%
No. of obs.	1,055,772	913,327	449,789	413,865

*Note:* Mean of the variables in our sample are reported in this table and calculations based on ACS 2010–18. Sample is restricted to those who are between 18 and 65, below 400% FPL, and not covered by VA Health Care or Indian Health Service. Other racial groups including American Indian, Native Hawaiian and other Pacific Islander and Alaska Native. All variables are binary except for Age, Household income and Family size, which are continuous. Calculations account for ACS sample weights.

### 4.2 Benchmark Results

**FIGURE 1:**
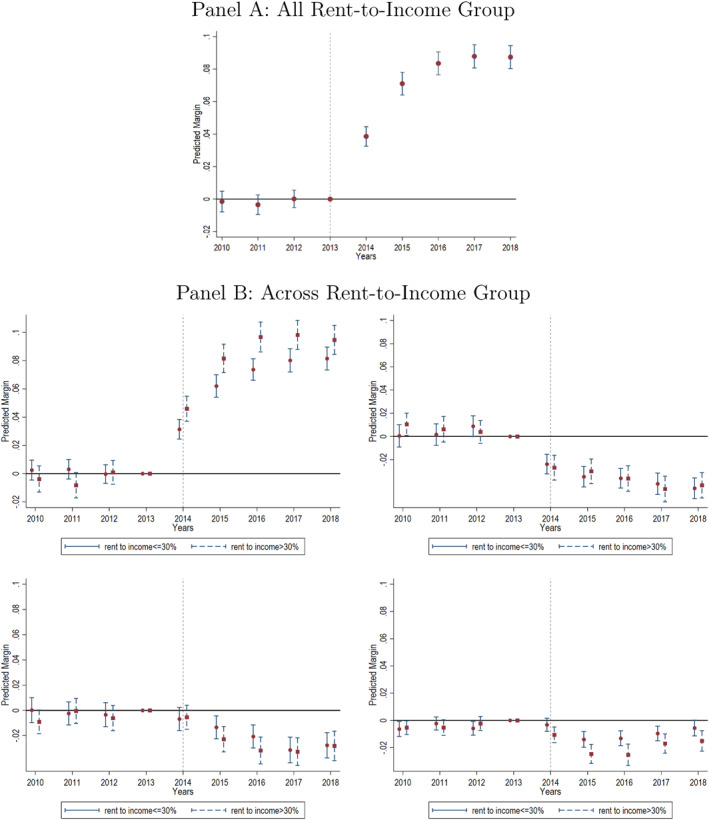
Estimated treatment effect on Medicaid coverage rate across year. The figure is based on a sample that excludes any early or late expansion states. The estimation is based on states that adopted Medicaid expansion in 2014 and those that did not participate. The red dots (or red squares) is the estimated average treatment effect on the non‐rent‐burdened group (or rent‐burdened group). The solid line (or dash line) is the 95% confidence interval for estimated average treatment effect on the non‐rent‐burdened group (or rent‐burdened group). Panel (A) shows the estimated treatment effect on Medicaid for households from all RTI groups; Panel (B), top left shows the predicted Medicaid coverage rate; top right shows the predicted uninsured rate; bottom left shows the predicted coverage rate on employer‐sponsored insurance; bottom right shows the predicted coverage rate on directly‐purchased insurance.

The key identifying assumption of our DID analysis is that the insurance coverage rates in both expansion and non‐expansion states follow the same trend in the absence of Medicaid expansion. The test of this parallel trend assumption for the full sample and each rent group are shown in event study analysis in Figure 1.^5^ Overall, we do not find supporting evidence that the trend of Medicaid coverage or other insurance coverage rates was different between expansion and non‐expansion states before the ACA went into effect.

Table 2 presents our DID results for Equation (1). We show the average coverage rate of different types of insurance during the pre‐expansion period in non‐expansion states and expansion states in columns (1) and (3) of Table 2, respectively. Columns (2) and (4) display the average coverage rate of different types of insurance during the post‐expansion period in non‐expansion states and expansion states, respectively.

**TABLE 2:** Average estimated treatment effect of 2014 Medicaid expansion on insurance coverage rates.

	States %					
	Non‐expansion		Expansion	
	Pre‐ACA 2010–2013 (1)	Post‐ACA 2015–2018 (2)	Pre‐ACA 2010–2013 (3)	Post‐ACA 2015–2018 (4)	Unadjusted difference‐in‐differences (standard error) (5)	Adjusted difference‐in‐differences (standard error) (6)
Panel A: Total sample						
Uninsured	32.30	22.10	24.70	11.35	$-3.40^{***}$ (0.35)	$-3.54^{***}$ (0.33)
Medicaid	18.89	19.49	26.15	34.73	$8.28^{***}$ (0.35)	$8.08^{***}$ (0.34)
Private						
Employer‐sponsored	40.28	46.35	42.49	45.86	$-2.99^{***}$ (0.31)	$-2.65^{***}$ (0.27)
Directly‐purchased	6.64	11.04	6.94	9.40	$-1.88^{***}$ (0.22)	$-1.86^{***}$ (0.22)
(Observations = 1,969,099)						
Panel B: Different RTI group						
Non‐rent‐burden group: RTI up to 30%						
Uninsured	29.01	20.64	22.96	11.38	$-3.68^{***}$ (0.34)	$-3.67^{***}$ (0.33)
Medicaid	13.48	14.59	18.84	26.63	$7.49^{***}$ (0.36)	$7.54^{***}$ (0.35)
Private						
Employer‐sponsored	49.97	54.32	52.74	55.22	$-2.40^{***}$ (0.35)	$-2.41^{***}$ (0.33)
Directly‐purchased	6.34	10.04	6.49	8.78	$-1.43^{***}$ (0.22)	$-1.42^{***}$ (0.22)
(Observations = 1,055,772)						
Rent‐burdened group: RTI above 30%						
Uninsured	35.96	23.95	26.56	11.30	$-3.13^{***}$ (0.45)	$-3.40^{***}$ (0.44)
Medicaid	24.91	25.72	33.93	44.46	$9.44^{***}$ (0.46)	$8.88^{***}$ (0.43)
Private						
Employer‐sponsored	29.48	36.22	31.59	34.62	$-3.81^{***}$ (0.46)	$-3.12^{***}$ (0.38)
Directly‐purchased	6.97	12.32	7.42	10.15	$-2.44^{***}$ (0.29)	$-2.37^{***}$ (0.29)
(Observations = 913,327)						

*Note:* The sample used in this analysis excludes five early expansion states (CA, MA, MN, WA, DC), as well as states that expanded after 2015 but before 2020 (AK, IN, LA, ME, MT, VA). Column (5) presents the unadjusted DID estimates without any household‐level controls. Column (6) presents the adjusted DID estimates, by controlling for respondents' age, sex, race, level of education, income‐to‐poverty ratio, citizenship status, marriage status, and employment status. We control for both year and state fixed effect in columns (5) and (6). The standard errors clustered at CPUMA level are in the parentheses.

***, ** and * indicate significance at levels 1%, 5%, and 10%.

With respect to the percentage of Medicaid coverage, expansion states gained significantly more. For example, before 2014, the average percentage of Medicaid coverage was 18.89% in non‐expansion states and 26.15% in expansion states. The rate of Medicaid increased by only 0.50% in non‐expansion states after 2014. However, among expansion states, the percentage of Medicaid coverage increased to 34.73% after 2014, about 8 pp higher than the rates of coverage before the Medicaid expansion was implemented.

Following Park et al. (2019), we use the DID model to estimate the treatment effect of Medicaid expansion on insurance coverage rate and use the year 2014 as a transition year. The estimated coefficient β in Equation (1) is shown in column (6) of Table 2. Column (5) lists the estimated treatment effect without household level controls $X_{\mathrm{ist}}$.

The estimates in panel A of Table 2 show that the treatment effect of Medicaid expansion on the percentage of Medicaid and overall insurance rate is statistically significant and economically larger in expansion states than in non‐expansion states. More specifically, it suggests that the adjusted increase in Medicaid coverage is 8.08 pp greater in expansion states than in non‐expansion states after the ACA went into effect. We find quantitatively similar results in column (5) where we do not control for household‐level demographic characteristics. Similarly, the treatment effect on the overall insurance coverage remains positive among expansion states because the uninsured rate decreased 3.54 pp more in expansion states after the ACA. In contrast to the positive treatment effects of the expansion of Medicaid, the employer‐sponsored insurance and directly‐purchased insurance rates significantly increased by 2.65 and 1.86 pp more in non‐expansion states than expansion states, respectively (see column (6) in panel A). Our results suggest that Medicaid expansion has a positive impact on health insurance coverage because it increases enrollment and reduces the need to purchase private insurance.

In Panel B of Table 2, we re‐estimate Equation (1) for two different RTI groups. We use the RTI ratio of 30% as a threshold to separate households into the rent‐burdened group (those above 30%) and the non‐rent‐burdened group. The treatment effect on the Medicaid take‐up rate is higher among the rent‐burdened group than those in the non‐rent‐burdened group. Adjusted Medicaid coverage rate increased by 7.54 pp more (95% confidence interval is [6.86 pp, 8.22 pp]) in expansion states than in non‐expansion states after the ACA implementation among those whose gross rent is up to 30%, whereas the difference is 8.88 pp (95% confidence interval is [8.02 pp, 9.74 pp]) in the rent‐burdened group. We also conduct similar regressions for high‐income household, that is, whose income level exceed 400% FPL. The regression results show that, as a result of the expansion, the Medicaid enrollment rate for high‐income individuals exhibits a slight improvement (1.76 pp for the non‐rent‐burdened group and 1.96 pp for the rent‐burdened group, respectively).^6^ These effects are much smaller than the treatment effects presented in Table 2. These findings align with our expectations since the policy targets individuals with incomes ranging from 100% to 400% FPL rather than high‐income individuals.

Our findings suggest that the higher Medicaid take‐up rate in the rent‐burdened group has a more pronounced effect on crowding out employer‐sponsored insurance and directly‐purchased insurance. In this group, the coverage rates for employer‐sponsored and directly‐purchased insurance experienced a larger decline compared to the non‐rent‐burdened group. The coverage rate of employer‐sponsored insurance and directly‐purchased insurance dropped by 3.12 and 2.37 pp more in non‐expansion states than expansion states in the rent‐burdened group, whereas they only dropped by 2.41 and 1.42 pp more in the non‐rent‐burdened group. Overall, the treatment effects on the uninsured rate are similar between the two groups.

### 4.3 Difference‐in‐Difference‐in‐Difference Results

Table 3 presents the estimation results for the triple difference model (2), with columns (1) through (4) displaying the respective outcomes. The second row measures the average treatment effect on insurance coverage for those in the non‐rent‐burdened group. The first row shows the estimated $\tilde{\beta }$, capturing the additional changes in insurance coverage after Medicaid expansion for those in the rent‐burdened group. The treatment effect on the Medicaid take‐up rate is positive and statistically significant. The pre‐post Medicaid coverage change in expansion states compared to non‐expansion states is 1.5 pp higher for the rent‐burdened group (column 2) than for the non‐rent‐burdened group. On the other hand, the pre‐post uninsured rate change in expansion‐versus non‐expansion‐states is 0.2 pp higher for individuals with a rent‐burdened relative to non‐rent‐burdened individuals, which is statistically insignificant (column 1). Similar to our DID results, the increase in Medicaid enrollment drives down the enrollment in employer‐sponsored insurance (column 3) and directly‐purchased coverage (column 4). The crowd‐out effects are slightly larger among those in the rent‐burdened groups. Though there is a significant difference in the treatment effect on the Medicaid take‐up rate between the rent‐burdened and the non‐rent‐burden group, no significant difference in the treatment effect on the uninsured rate is observed between these two groups.

**TABLE 3:** Triple difference regression results.

	All income level				Income above 100% FPL			
	Uninsured	Medicaid	Employer	Direct	Uninsured	Medicaid	Employer	Direct
	(1)	(2)	(3)	(4)	(5)	(6)	(7)	(8)
HighRent×Expanded×Post	0.002	$0.015^{***}$	$-0.007^{*}$	$-0.010^{**}$	0.007	$0.015^{***}$	−0.008	$-0.015^{**}$
	(0.004)	(0.004)	(0.004)	(0.003)	(0.004)	(0.004)	(0.005)	(0.003)
Expanded×Post	$-0.037^{**}$	$0.075^{***}$	$-0.024^{**}$	$-0.014^{**}$	$-0.033^{***}$	$0.071^{***}$	$-0.025^{**}$	$-0.013^{**}$
	(0.003)	(0.003)	(0.003)	(0.002)	(0.003)	(0.003)	(0.003)	(0.002)
No. of observations	1,969,099	1,969,099	1,969,099	1,969,099	1,490,705	1,490,705	1,490,705	1,490,705
Year FE	Yes	Yes	Yes	Yes	Yes	Yes	Yes	Yes
State FE	Yes	Yes	Yes	Yes	Yes	Yes	Yes	Yes

*Note:* The sample used in this analysis excludes five early expansion states (CA, MA, MN, WA, DC), as well as states that expanded after 2015 but before 2020 (AK, IN, LA, ME, MT, VA). Columns (1) to (4) are estimated coefficients based on full samples. Columns (5) to (8) are estimated coefficients based on the subsample with annual income above 100 FPL. Other controls in Equation (2) are included but not reported. Dynamic triple difference regression results are presented in Supporting Information S1: Table D2, and the complete triple difference regression results can be found in Supporting Information S1: Table D3. The standard errors clustered at CPUMA level are in the parentheses.

***, ** and * indicate significance at levels 1%, 5%, and 10%.

Because of expanded Medicaid coverage for people with annual incomes of up to 138% FPL and subsidized private coverage purchased via Marketplaces for people with incomes of 138%–400% FPL who weren't offered affordable coverage through an employer, we re‐run Equation (2) based on a sub‐sample for those with an annual income between 100% and 400% FPL. The estimation results are presented in columns (5) through (8) in Table 3. The estimated coefficients are qualitatively and quantitatively similar to those which we obtained by using the full sample. The treatment effects we observe in our full sample analysis are mostly driven by the treatment effects among households whose annual income is above 100% FPL. These households are expected to benefit from Medicaid expansion.

### 4.4 Event Study Results

Similar to the methodology used in Miller, Johnson, and Wherry (2021), we use the estimation as our Equation (3) and present the estimated evolution of the treatment effect for Medicaid expansion in 2014 in Figure 1. Our parallel testing results suggest no significant difference in individuals' take‐up rates between expansion and non‐expansion states (*p*‐value = 0.56), regardless of their RTI ratio. Similar to our previous findings, panel A of Figure 1 shows that the treatment effect is significantly greater than zero in the transition year of 2014, though quantitatively smaller than the treatment effect in later years after the expansion was fully implemented. The treatment effect from the year 2015 to the year 2018 is 8.11 pp on average, which is close to our overall DID estimates (i.e., 8.08 pp) in Equation (1) and Table 2. Following Goodman‐Bacon (2021) and Miller, Johnson, and Wherry (2021), we compare our estimates in the DID approach to the average coefficient in post‐ACA from the event study. Our findings suggest that these estimates are similar, which implies that the time‐varying treatment effects do not bias our DID results. We also estimate the evolution of the treatment effect for the later expansion states.^7^ We find that the average effects are 5.37 pp for expansion in 2015 and 13.93 pp for expansion in 2016. The average treatment effects computed from the event study are quantitatively similar to the treatment effect estimated using our DID model in Equation (1), that is, 5.41 and 13.67 pp.

Panel B of Figure 1 illustrates the difference in treatment effect between non‐rent‐burdened households and rent‐burdened households. In the transition year of 2014, the estimated take‐up rate is 3.23% among individuals in the non‐rent‐burdened group, which is lower than the take‐up rate of 4.53% among individuals in the rent‐burdened group. However, the difference between these two treatment effects is not significant in the transition year. In subsequent years, the difference in the treatment effect enlarged, becoming significant in 2015 and 2016, but then it shrunk and became insignificant in 2017 and 2018. Compared to the non‐rent‐burdened group, Medicaid coverage rate additionally increases by 1.43 pp in 2015 and 1.80 pp in 2016 among the rent‐burdened households. Our estimation shows again that Medicaid expansion has a larger treatment effect for rent‐burdened households, with the largest additional treatment effect observed 2 years after the expansion.

Despite the significant difference in the treatment effect on Medicaid coverage rate between the rent‐burdened and non‐rent‐burdened group, we do not find any significant difference in the uninsured rate and other insurance coverage rates (i.e., employer‐sponsored and directly‐purchased) between these two groups across years. Similar to our DDD findings in Table 3, Medicaid expansion drives down enrollment in employer‐sponsored insurance and directly‐purchased coverage. But the crowd‐out effects are smaller than the positive treatment effect on Medicaid enrollment, resulting in a negative treatment effect on the uninsured rate. The treatment effect on other insurance coverage is not significantly different from zero in the transition year. In addition, the treatment effect on directly‐purchased insurance becomes less negative 2 years after Medicaid expansion.

### 4.5 Robustness Check

To assess the robustness of our findings on Medicaid take‐up and uninsured rates across different RTI groups, we conducted several additional checks on a subsample of household with annual income above 100% of FPL, that is those benefits from ACA expansion on Medicaid eligibilities. The results from these checks align closely with those from our benchmark estimates, suggesting that our initial findings are robust and reflect the direct impact of Medicaid expansion rather than being driven by specific sample characteristics or other provisions of the ACA.

#### 4.5.1 Early ACA Provision and Age Group

Firstly, we examined the impact of the early ACA provision that allows young adults to remain on their parents' insurance plans until age 26. This provision could potentially influence the Medicaid take‐up rates among different age groups, as younger adults eligible for Medicaid might choose to stay on their parents' plans instead of enrolling in Medicaid (Sommers et al. 2013; Hamilton 2024). To explore this, we compared Medicaid take‐up rates between individuals younger than 26 and those older than 26. Our hypothesis was that individuals above 26 would exhibit higher Medicaid take‐up rates due to the lack of an alternative insurance option. The regression results, presented in columns (1) to (4) of Table 4, support this hypothesis: among individuals older than 26, the pre‐post Medicaid coverage change in expansion states compared to non‐expansion states is 1.7 pp more for the rent‐burdened group, which is 1.3 pp higher for individuals up to 26. These findings indicate that while the ACA's Medicaid expansion has significantly impacted older adults, younger adults may not rely on it due to alternative insurance options available through their parents. This suggests that Medicaid policy outreach might be more effectively targeted toward older adults who lack these alternatives.

**TABLE 4:** Robustness: Early ACA provision.

	Above age 26		Up to age 26		Drop year 2010		Drop year 2010, 2011	
	Uninsured	Medicaid	Uninsured	Medicaid	Uninsured	Medicaid	Uninsured	Medicaid
	(1)	(2)	(3)	(4)	(5)	(6)	(7)	(8)
HighRent×Expanded×Post	$0.009^{*}$	$0.017^{***}$	−0.000	$0.013^{*}$	−0.000	$0.019^{***}$	0.000	$0.022^{***}$
	(0.005)	(0.005)	(0.008)	(0.007)	(0.004)	(0.004)	(0.005)	(0.004)
Expanded×Post	$-0.033^{***}$	$0.071^{***}$	$-0.034^{***}$	$0.070^{***}$	$-0.039^{***}$	$0.081^{***}$	$-0.039^{***}$	$0.080^{***}$
	(0.003)	(0.003)	(0.006)	(0.005)	(0.004)	(0.003)	(0.004)	(0.003)
No. Observations	1,099,212	1,099,212	391,493	391,493	1,702,622	1,702,622	1,500,912	1,500,912
Year FE	Yes	Yes	Yes	Yes	Yes	Yes	Yes	Yes
State FE	Yes	Yes	Yes	Yes	Yes	Yes	Yes	Yes

*Note:* The sample used in this analysis are household with annual income above 100 FPL, and excludes five early expansion states (CA, MA, MN, WA, DC), as well as states that expanded after 2015 but before 2020 (AK, IN, LA, ME, MT, VA). Other controls in equation are included but not reported. The standard errors clustered at CPUMA level are in the parentheses.

***, ** and * indicate significance at levels 1%, 5%, and 10%. Results on changes in other insurance coverage can be found in Supporting Information S1: Tables E1–E3.

Though this provision on dependent coverage went into effect in 2010, to ensure that the including data from 2010 to 2011 did not introduce potential bias in our analysis, we conducted robustness checks by excluding the year 2010 and, separately, both 2010 and 2011, from the analysis. The regression results, presented in columns (5) to (8) of Table 4, demonstrate that our findings remain robust regardless of the starting year. This consistency confirms that the observed effects of Medicaid expansion on health insurance coverage and housing affordability are not driven by early ACA provisions or changes in the sample composition during these initial years.

#### 4.5.2 Different Rent‐to‐Income Threshold

We also conducted robustness checks to verify the stability of our findings across various definitions of rent‐burdened households, following U.S. Department of Housing and Urban Development (HUD) (2024) definition of a rent‐burdened household as one spending more than 30% of its income on rent. To test the robustness of our results, we applied alternative thresholds—40%, 50%, and even 60% of income.^8^ The regression results, presented in columns (1) to (6) of Table 5, reveal no significant differences in our triple‐difference estimates on Medicaid take‐up rates across these thresholds.^9^ Consistently with our benchmark analysis, households defined as rent‐burdened at the 40%, 50%, and 60% thresholds show a significant increase in Medicaid enrollment compared to the non‐rent‐burden group.

**TABLE 5:** Robustness: Different rent‐to‐income threshold.

	Threshold: 40%		Threshold: 50%		Threshold: 60%		Continuous RTI	
	Uninsured	Medicaid	Uninsured	Medicaid	Uninsured	Medicaid	Uninsured	Medicaid
	(1)	(2)	(3)	(4)	(5)	(6)	(7)	(8)
HighRent×Expanded×Post	0.007	$0.023^{***}$	0.009	$0.024^{***}$	0.017	$0.023^{**}$		
	(0.005)	(0.005)	(0.008)	(0.007)	(0.011)	(0.010)		
Expanded×Post	$-0.031^{***}$	$0.072^{***}$	$-0.031^{***}$	$0.074^{***}$	$-0.031^{***}$	$0.075^{***}$	$-0.031^{***}$	$0.056^{***}$
	(0.003)	(0.003)	(0.003)	(0.003)	(0.003)	(0.003)	(0.004)	(0.005)
RTI×Expanded×Post							0.018	$0.045^{***}$
							(0.014)	(0.013)
No. Observations	1,490,705	1,490,705	1,490,705	1,490,705	1,490,705	1,490,705	1,490,705	1,490,705
Year FE	Yes	Yes	Yes	Yes	Yes	Yes	Yes	Yes
State FE	Yes	Yes	Yes	Yes	Yes	Yes	Yes	Yes

*Note:* The sample used in this analysis are household with annual income above 100 FPL, and excludes five early expansion states (CA, MA, MN, WA, DC), as well as states that expanded after 2015 but before 2020 (AK, IN, LA, ME, MT, VA). Other controls in equation are included but not reported. The standard errors clustered at CPUMA level are in the parentheses.

∗**, ** and * indicate significance at levels 1%, 5%, and 10%. Results on changes in other insurance coverage can be found in Supporting Information S1: Tables E4 and E5.

Additionally, the regression analysis in columns (7) to (8) of Table 5 replaces the dummy variable for rent‐burdened with a continuous rent‐to‐income ratio. The results demonstrate a significant positive association between the rent‐to‐income ratio and Medicaid take‐up rates post‐expansion: in expansion states, a 1% increase in the rent‐to‐income ratio corresponds to a further increase of 0.045 pp in Medicaid enrollment. The results from these tests, which are qualitatively similar across all thresholds, suggest that the benefits of the ACA expansion extend robustly to households experiencing different degrees of rent‐induced financial pressure.

#### 4.5.3 State‐Level Policies

In our third robustness check, we assessed the potential influence of other state‐level policies on our benchmark results. First, we add state‐by‐year fixed effects into our regression models to control for unobserved changes in state policies. The estimation results in columns (1) and (2) of Table 6 shows that Medicaid enrollment for individuals in rent‐burdened households remains significantly increased by 1.9 pp, consistent with our initial findings. Additionally, the results indicate that employer‐sponsored coverage and uninsured rates are not significantly affected by these state‐level changes during the study period.

**TABLE 6:** Robustness: State policies.

	State‐year FE		Drop waiver states		Within state‐run		Drop state‐run	
	Uninsured	Medicaid	Uninsured	Medicaid	Uninsured	Medicaid	Uninsured	Medicaid
	(1)	(2)	(3)	(4)	(5)	(6)	(7)	(8)
HighRent×Expanded×Post	−0.003	$0.019^{***}$	0.006	$0.015^{**}$	−0.001	$0.018^{***}$	$-0.027^{***}$	$0.022^{***}$
	(0.004)	(0.004)	(0.006)	(0.006)	(0.004)	(0.004)	(0.006)	(0.006)
Expanded×Post	$-0.169^{***}$	$0.125^{***}$	$-0.033^{***}$	$0.071^{***}$	$-0.152^{***}$	$0.125^{***}$	$-0.153^{***}$	$0.140^{***}$
	(0.027)	(0.014)	(0.007)	(0.006)	(0.054)	(0.014)	(0.011)	(0.019)
No. Observations	1,490,705	1,490,705	1,403,604	1,403,604	261,367	261,367	1,229,338	1,229,338
Year FE	Yes	Yes	Yes	Yes	Yes	Yes	Yes	Yes
State FE	Yes	Yes	Yes	Yes	Yes	Yes	Yes	Yes
State‐year FE	Yes	Yes	No	No	No	No	No	No

*Note:* The sample used in this analysis are household with annual income above 100 FPL, and excludes five early expansion states (CA, MA, MN, WA, DC), as well as states that expanded after 2015 but before 2020 (AK, IN, LA, ME, MT, VA). Waiver States are AR, IN, IA, MI. States that has their own state‐run programs are CA, CO, CT, DC, MD, MA, MN, NV, NY, RI, and VT. Other controls in equation are included but not reported. The standard errors clustered at CPUMA level are in the parentheses.

***, ** and * indicate significance at levels 1%, 5%, and 10%. Results on changes in other insurance coverage can be found in Supporting Information S1: Table E6.

We also examine the impact of ACA expansion on waiver states (i.e. Arkansas, Indiana, Iowa and Michigan), which implemented additional Medicaid programs such as work requirements, premiums, or co‐pays. Excluding waiver states in our analysis (columns (3) and (4) of Table 6), we found that Medicaid enrollment continues to show a significant increase of 1.5 pp for individuals in rent‐burdened households. While waivers introduce specific eligibility conditions, they do not fundamentally change the overall positive impact of Medicaid expansion on coverage and insured rates.

Furthermore, we explored the differential effects of states operating their own private insurance exchanges versus those using federal exchanges. Two additional checks were performed: one within states with their own exchanges^10^ (columns (5) and (6) of Table 6) and another analyzing excluding states with state‐run exchanges (columns (7) and (8) of Table 6). Results from columns (7) and (8) show that excluding state‐run exchanges, Medicaid enrollment still significantly increased by 2.2 pp among rent‐burdened households. In states with state‐run exchanges, Medicaid enrollment saw a significant rise of 1.8 pp, with no significant shifts in uninsured rates. These findings indicate that while state‐run exchanges might slightly influence private insurance outcomes due to administrative differences or outreach efforts, the core impact of Medicaid expansion on public insurance coverage remains consistent, irrespective of the type of exchange.

#### 4.5.4 Childcare Cost and Housing Related Expenses

In the fourth set of robustness checks, we considered additional household expenses other than rent, which could also influence financial well‐being. Expenses such as childcare costs and essential utilities are inflexible and may limit health‐related expenditures. To better account for these costs, we include the following variables in our regressions: (1) the number of children in the household as a proxy for childcare costs (see column (1) and (2) of Table 7); (2) household‐level costs such as electricity, fuel, gas, and water expenses (see column (3) and (4) of Table 7). The inclusion of these variables does not significantly alter our primary results. As shown in columns (1) to (4) of Table 7, the increase in Medicaid take‐up rate for rent‐burdened households remains statistically significant, as does the observed decrease in direct‐purchase insurance coverage. Furthermore, the coefficient on the number of children is significant across all types of insurance coverage, indicating that having more children in a household increases the likelihood of Medicaid enrollment while simultaneously reduces the likelihood of being uninsured or relying on direct‐purchase insurance. However, they do not fundamentally change the differential impact of Medicaid expansion between rent‐burdened and non‐rent‐burdened households. Hence, our main findings of the effects of rent burdens on health insurance uptake under the ACA expansion remain robust even when considering these additional household expenses.

**TABLE 7:** Robustness: Childcare costs and alternative measures.

	Childcare costs		Housing expenses		Overcrowding		High mortgage	
	Uninsured	Medicaid	Uninsured	Medicaid	Uninsured	Medicaid	Uninsured	Medicaid
	(1)	(2)	(3)	(4)	(5)	(6)	(7)	(8)
HighRent×Expanded×Post	0.006	$0.015^{***}$	0.006	$0.013^{**}$				
	(0.004)	(0.004)	(0.006)	(0.006)				
Expanded×Post	$-0.033^{***}$	$0.071^{***}$	$-0.035^{***}$	$0.079^{***}$	$-0.031^{**}$	$0.069^{***}$	$-0.019^{**}$	$0.051^{***}$
	(0.003)	(0.003)	(0.004)	(0.004)	(0.003)	(0.003)	(0.003)	(0.003)
NumberofChildren	$-0.017^{***}$	$0.030^{***}$						
	(0.001)	(0.001)						
Overcrowd×Expanded×Post					0.003	$0.013^{***}$		
					(0.005)	(0.004)		
HighMortgage×Expanded×Post							$0.007^{**}$	$0.009^{***}$
							(0.003)	(0.003)
No. Observations	1,490,705	1,490,705	773,283	773,283	1,490,705	1,490,705	2,803,259	2,803,259
Year FE	Yes	Yes	Yes	Yes	Yes	Yes	Yes	Yes
State FE	Yes	Yes	Yes	Yes	Yes	Yes	Yes	Yes

*Note:* The sample used in this analysis are household with annual income above 100 FPL, and excludes five early expansion states (CA, MA, MN, WA, DC), as well as states that expanded after 2015 but before 2020 (AK, IN, LA, ME, MT, VA). Other controls in equation are included but not reported. The standard errors clustered at CPUMA level are in the parentheses.

***, ** and * indicate significance at levels 1%, 5%, and 10%. Results on changes in other insurance coverage can be found in Supporting Information S1: Tables E7–E10.

#### 4.5.5 Other Robustness Check

In our final set of robustness checks, we consider alternative measures that could affect health conditions through housing‐related financial stress. Other factors of affordability and stability, such as overcrowding and high mortgage payments, can significantly influence individuals' health insurance enrollment outcomes (see Table 7). Overcrowding, defined as having more than one person per bedroom (the median number of people per bedroom is one in our data set). In columns (5) and (6) in Table 7 replace the high rent dummy with an overcrowding dummy. Our results show that, after the year 2014, in states that expanded Medicaid, there was an estimated 1.3 pp additional increase in Medicaid coverage among those living in overcrowded households. This suggests that overcrowding may exert financial pressures that affect health insurance enrollment decisions, similar to rent burdens.

In addition to renters, homeowners facing substantial mortgage payments also represent another critical facet of housing affordability. We introduced a “High Mortgage” dummy variable, defined as households spending more than 22% of their income on mortgage payments. As shown in columns (7) and (8) of Table 7, the pre‐post Medicaid coverage change in expansion states compared to non‐expansion states is 0.9 pp more for the high mortgage group. In comparison to renters, individuals with high mortgage costs experienced the same increase in Medicaid coverage and a similar decrease in directly‐purchased insurance. Further statistical testing is needed to precisely quantify and confirm any significant distinctions between the two groups' responses to housing‐related financial challenges.

Finally, we address concerns related to clustering, the inclusion of the early expansion states, and specific year exclusions (see Table 8). First, we cluster standard errors at the state level to account for state‐specific shocks, and the results remain consistent with our primary findings (columns (1) and (2) of Table 8). Second, we include early expansion states to ensure that these states do not disproportionately affect the results. Our findings, as shown in columns (3) and (4), remain robust. Third, we include the years 2008 and 2009 to capture pre‐expansion trends more comprehensively. As indicated in columns (5) and (6), our main results remain unaffected. Finally, to address concerns that 2014 might introduce noise due to staggered implementation of the ACA provisions, we exclude this year, and the results shown in columns (7) and (8) continue to support our primary conclusions. These checks further validate the robustness and reliability of our findings across various model specifications and alternative measures.

**TABLE 8:** Robustness: others.

	Clustering at state		Add early expansion		Add years 2008 & 2009		Exclude year 2014	
	Uninsured	Medicaid	Uninsured	Medicaid	Uninsured	Medicaid	Uninsured	Medicaid
	(1)	(2)	(3)	(4)	(5)	(6)	(7)	(8)
HighRent×Expanded×Post	0.006	$0.015^{**}$	0.004	$0.020^{***}$	0.004	$0.017^{***}$	0.006	$0.015^{***}$
	(0.006)	(0.006)	(0.004)	(0.004)	(0.004)	(0.004)	(0.004)	(0.004)
Expanded×Post	$-0.033^{***}$	$0.071^{***}$	$-0.040^{***}$	$0.081^{***}$	$-0.029^{***}$	$0.073^{***}$	$-0.033^{***}$	$0.071^{***}$
	(0.007)	(0.006)	(0.004)	(0.003)	(0.003)	(0.003)	(0.003)	(0.003)
No. Observations	1,490,705	1,490,705	1,901,379	1,901,379	1,780,643	1,780,643	1,321,608	1,321,608
Year FE	Yes	Yes	Yes	Yes	Yes	Yes	Yes	Yes
State FE	Yes	Yes	Yes	Yes	Yes	Yes	Yes	Yes

*Note:* The sample used in this analysis are household with annual income above 100 FPL, and excludes five early expansion states (CA, MA, MN, WA, DC), as well as states that expanded after 2015 but before 2020 (AK, IN, LA, ME, MT, VA). Other controls in equation are included but not reported. The standard errors clustered at CPUMA level are in the parentheses.

***, ** and * indicate significance at levels 1%, 5%, and 10%. Results on changes in other insurance coverage can be found in Supporting Information S1: Tables E11–E14.

## 5 Discussion

The financial strain caused by high housing costs significantly increases the need for Medicaid coverage because these expenses reduce households' flexibility to afford other essentials, such as healthcare. Ali, Bradford, and Maclean (2024) briefly mentions the relationship between housing instability and insurance coverage. Our study extends this by examining how households with higher RTI ratios were disproportionately impacted by financial constraints, leading to lower health insurance coverage prior to the expansion. Medicaid expansion alleviated these unmet needs more effectively for those in rent‐burdened households than private or employer‐sponsored insurance, which often involves higher premiums or co‐pays. Our findings demonstrate how Medicaid expansion specifically benefits those who experience both healthcare and housing instability, as it serves as an essential safety net for these vulnerable populations.

This study makes several important contributions to the literature on Medicaid expansion, particularly at the intersection of healthcare insurance and housing affordability. First. previous studies have shown that Medicaid expansion increases health insurance coverage among low‐income populations (Sommers et al. 2015; Courtemanche et al. 2017; Kaestner et al. 2017; Chu 2023). Our study extends this body of work by focusing on rent‐burdened households, who face compounded financial pressure due to high housing costs and inadequate healthcare coverage. Our findings suggest that Medicaid expansion plays a key role in reducing out‐of‐pocket healthcare costs for rent‐burdened households, making it a more effective alternative than direct‐purchased or employer‐sponsored insurance. This is especially important for low‐income, rent‐burdened households, where the financial pressure from both housing costs and healthcare expenses is most intense.

Second, our study contributes to the ongoing discussion on the “crowding‐out” effect of Medicaid. While private insurance did increase in non‐expansion states more, our DiD and triple difference models control for state‐level fixed effects and time trends, allowing us to separate the effects of Medicaid expansion from broader market shifts. The decrease in employer‐sponsored insurance in expansion states is attributed primarily to the crowding‐out effect of Medicaid, which offers more affordable coverage for rent‐burdened households. Our robustness checks confirm that this crowding‐out effect remains significant, demonstrating that the shift away from employer‐sponsored insurance in expansion states is not simply a result of broader trends in private insurance. Additionally, by mitigating “job‐lock,” where individuals remain in jobs solely for health insurance, Medicaid expansion fosters labor market flexibility (Madrian 1994; Hamersma and Kim 2009; Bae, Meckel, and Shi 2022). This perspective enhances our understanding of Medicaid's broader impacts on both the workforce and insurance markets.

Furthermore, our research contributes to the understanding of how different dimensions of vulnerability, including housing affordability, influence Medicaid take‐up. Prior studies observed heterogeneity in Medicaid expansion outcomes across various vulnerabilities, such as minority groups (Ye and Rodriguez 2021), low‐income parents (McMorrow et al. 2017) and children in low‐income families (Hudson and Moriya 2017; Lombardi, Bullinger, and Gopalan 2022; Soni, Wherry, and Simon 2020). By adding housing affordability as another key dimension of vulnerability, our research shows how Medicaid alleviates financial strain for rent‐burdened households. To further improve Medicaid enrollment for these populations, policies should streamline the application process and enhance outreach efforts, as highlighted in previous expansions studies (Hudson and Moriya 2017, 2020). Addressing these barriers will ensure that vulnerable households can obtain the heath insurance they need.

From a policy perspective, it is crucial to focus on transitional assistance programs and targeted outreach efforts to mitigate the impact of Medicaid unwinding, especially for the groups that benefited most from the expansion, such as rent‐burdened and low‐income households. These populations are particularly vulnerable to the rollback of Medicaid eligibility, as they already face significant financial challenges related to housing affordability. Policymakers should consider integrating housing affordability into Medicaid policy design, such as offering rent subsidies or expanding Medicaid eligibility in high‐cost areas, especially for those who are not eligible for housing vouchers, often experience severe financial burdens. Low‐income individuals may qualify for Medicaid but lose eligibility if their income slightly exceeds the threshold, forcing them to pay more for private insurance. Additionally, addressing barriers to Medicaid enrollment, including stigma and administrative hurdles (Allen et al. 2014), could improve participation and ensure that these vulnerable households continue to access insurance.

Our study has two limitations. First, while the ACS provides valuable data on housing expenses and insurance coverage, it lacks detailed information on other significant financial burdens, such as childcare costs and household debt. This limits our ability to fully capture the broader financial pressures faced by rent‐burdened households. Future research could explore how additional financial constraints interact with Medicaid expansion using datasets that include comprehensive financial information. Second, although our models control for state‐level fixed effects and time trends, unobserved factors in non‐expansion states may influence the observed crowding‐out effect of Medicaid. Additionally, although previous research has shown that having insurance coverage increases access to care and improves health outcomes (Sommers, Gawande, and Baicker 2017a), future research could use longitudinal data to examine the long‐term impacts of Medicaid expansion on health outcomes and socioeconomic mobility, especially across different levels of housing affordability.

## Ethics Statement

The authors have nothing to report.

## Conflicts of Interest

The authors declare no conflicts of interest.

## Supporting information

Supporting Information S1

## Data Availability

The data that support the findings of this study are available in U.S. Census FTP Repository for ACS Data at https://www2.census.gov/programs‐surveys/acs/data/. These data were derived from the following resources available in the public domain: ‐ American Community Survey, https://www.census.gov/programs‐surveys/acs/data.html.

## Endnotes

^1^
The U.S. Department of Housing and Urban Development (HUD) defines rent‐burdened families are those spending more than 30% of their income on rents (U.S. Department of Housing and Urban Development (HUD), 2024) and severe rent‐burdened are those spending more than 50% of their income.

^2^
According to Kaiser Family Foundation (2023), five states (California, District of Columbia, Massachusetts, Minnesota, Washington) expanded eligibility before 2014. Six states (Alaska, Indiana, Louisiana, Maine, Montana, Virginia) expanded eligibility between 2015 and 2018. The rest expanded in 2014: Arizona, Arkansas, Colorado, Connecticut, Delaware, Hawaii, Illinois, Iowa, Kentucky, Maryland, Michigan, Nevada, New Hampshire, New Jersey, New Mexico, New York, North Dakota, Ohio, Oregon, Pennsylvania, Rhode Island, Vermont, and West Virginia.

^3^
We cluster standard errors at state level as a robustness check. Regression results in columns (5) and (6) of Table 7 remain consistent with benchmark analysis.

^4^
As an extension, we also evaluate the effect of Medicaid expansion which was implemented in 2015 and 2016. Results are available in our online Appendix.

^5^
We also directly test the parallel trend assumption using pre‐expansion sample, and the results are in Supporting Information S1: Table D1

^6^
Regression results can be found in Supporting Information S1: Table A2

^7^
Later expansion states are those that implemented Medicaid expansion in 2015 and 2016: Alaska, Indiana, Louisiana, Maine, Montana, Virginia. Results can be found in Supporting Information S1: Table C2, Figures C1, C2.

^8^
The distribution of rent‐to‐income ratios can be found in Supporting Information S1: Figure A1.

^9^
In our online appendix, we run regression on a subgroup of households spending more than 30% of their income on rents, and examine whether there's significant difference in the treatment effect between those rent‐burdened and server rent‐burdened. The regression results in Supporting Information S1: Table E5, indicate that higher housing expenditure is associated with an increase in Medicaid take‐up rates following ACA expansions, although the differences are not statistically significant

^10^
States that has their own state‐run programs are California, Colorado, Connecticut, District of Columbia, Maryland, Massachusetts, Minnesota, Nevada, New York, Rhode Island and Vermont.
