# Supplementary material for: Health Insurance Coverage Changes Under the Affordable Care Act Among High Housing Cost Households, 2010–18
Source: Health Econ. 2024 Nov 13;34(3):415–30. doi: 10.1002/hec.4912 (PMC11786949; doi:10.1002/hec.4912)
Supplement: Supplementary file 1 — Supporting Information S1 [file HEC-34-415-s001.pdf]

Appendix

A DID and Income-to-poverty Ratio

We could separate our data by individuals' income-to-poverty ratio to control for the effects of individuals' wealth, which highly influences their decision-making in rent and Medicaid take-up. Following the definition of income-to-poverty ratio, individuals could be separated into less or equal to 100% FPL (*Below100% FPL*) or higher than 100% but less or equal to 400% FPL (*Above100% FPL*). In Table A1, we present the summary statistics and regression results of non-rent-burden households by classifying their income-to-poverty ratio separately (i.e., *Below100% FPL* and *Above100% FPL*) in the Panel A. We also provide similar information for rent-burdened households based on their income-to-poverty ratio in Panel B.

By making comparisons on treatment effects within each panel, we find that *Below100% FPL* individuals have a significantly higher improvement rate than *Above100% FPL* individuals. This effect holds for both non-rent-burdened and rent-burdened households. More specifically, in Panel A (non-rent-burden households), Medicaid expansion leads to a 11.23 pp increase in the rate of Medicaid in expansion states over non-expansion states, while the treatment effects on individuals in rent-burdened households are merely 7.10 pp. In Panel B (rent-burdened households), the ACA leads to a 10.24 pp increase in Medicaid coverage in expansion states over non-expansion states, while the treatment effects on those in rent-burdened households are merely 8.42 pp.

The total sample observations of non-rent-burdened and rent-burdened groups are similar (i.e., 1,055,772 observations in Panel A and 913,327 observations in Panel B). we find that the number of observations of income ratio groups within different level of rent burdens matters for Medicaid coverage. For the non-rent-burdened group, there are 932,984 out of 1,055,772 (or 88.37%) individuals in the group of *Above100% FPL* (the less affected group compared to the group of *Below100% FPL*) for their income-to-poverty ratio. On the contrary, for those with a rent-burden group (*RTI above 30%*), 557,721 out of 913,327 (or 61.07%) individuals are in the group of *Above100% FPL*, and they have 8.42 pp of improvement in the rate

Medicaid.

Our findings suggest that, not surprisingly, there is a relatively higher percentage of people among the *Below100% FPL* income-to-poverty ratio in the rent-burdened group than in the non-rent-burdened group, resulting in the former group benefiting more from the expansion. Using the same interpretation, we show that the difference in observations between FPL helps us understand that the crowd-out effect is slightly larger among those in the rent-burdened group than in the non-rent-burdened group.

Figure A1: Distribution of Rent-to-Income Ratio among household in each income bracket

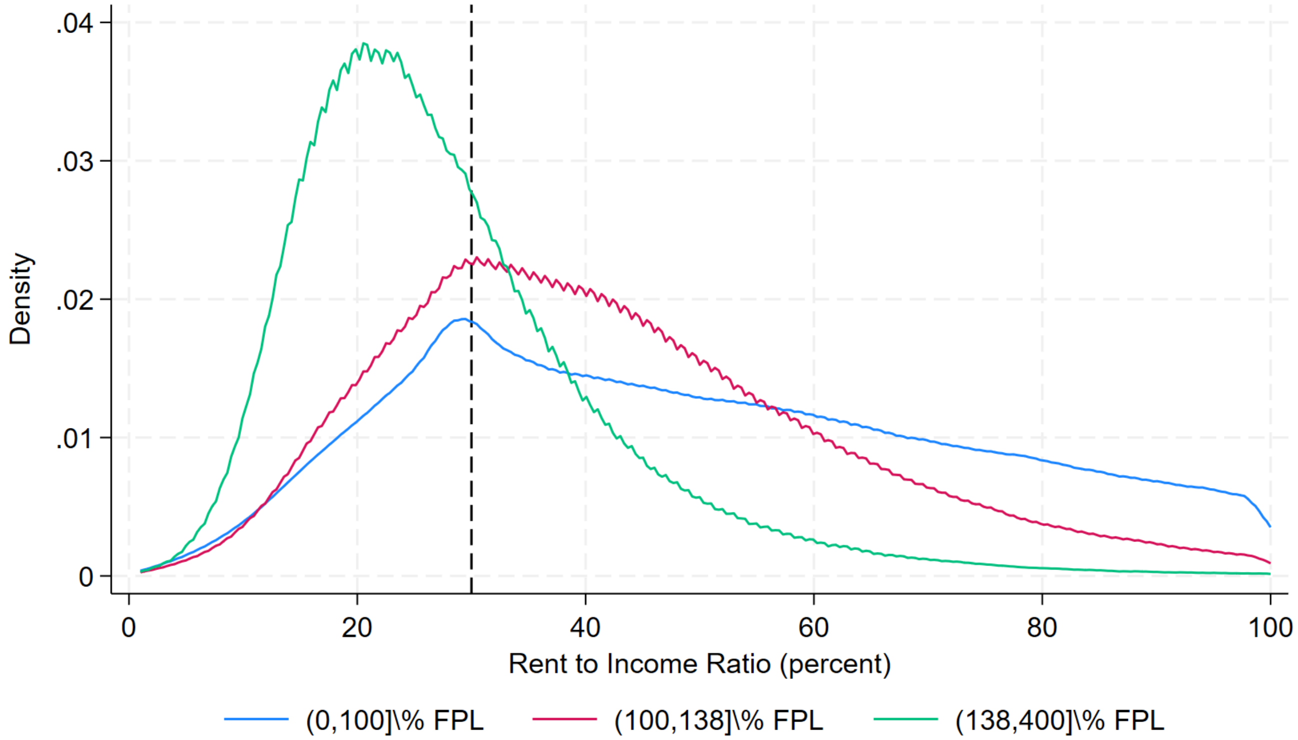

Notes: The charts plots the distribution of rent-to-income ratio for three group of household: low-income household that are below 100% FPL, low-income household that between 100% and 138% FPL, low-to-medium income household that between 138% and 400% FPL.

Table A1: Average Estimated Treatment Effect of 2014 Medicaid Expansion on Insurance Coverage Rates (Separate by Income-to-Poverty Ratio)

	States %							
	Non-Expansion		Expansion		Unadjusted Difference-in-Differences (standard error)		Adjusted Difference-in-Differences (standard error)	
	Pre-ACA 2008-2013	Post-ACA 2015-2018	Pre-ACA 2008-2013	Post-ACA 2015-2018				
	(1)	(2)	(3)	(4)	(5)		(6)	
Panel A: RTI up to 30%								
Below100% FPL								
Uninsured	40.64	29.92	28.89	11.77	−6.33***	(0.87)	−6.58***	(0.83)
Medicaid	37.74	39.67	50.12	62.68	11.32***	(1.01)	11.23***	(0.92)
Private								
Employer-sponsored	15.95	21.46	15.61	19.36	−2.76***	(0.77)	−2.47***	(0.70)
Directly-purchased	5.73	9.69	6.80	8.36	−2.50***	(0.47)	−2.43***	(0.46)
(Observations = 122,788)								
Above100% FPL								
Uninsured	27.51	19.56	22.10	11.33	−3.31***	(0.33)	−3.27***	(0.32)
Medicaid	10.34	11.67	14.34	21.77	7.01***	(0.34)	7.10***	(0.33)
Private								
Employer-sponsored	54.38	58.15	58.09	60.05	−2.40***	(0.37)	−2.48***	(0.34)
Directly-purchased	6.42	10.08	6.45	8.83	−1.28***	(0.23)	−1.27***	(0.23)
(Observations = 932,984)								
Panel B: RTI above 30%								
Below100% FPL								
Uninsured	39.18	28.15	26.49	9.93	−5.40***	(0.64)	−5.59***	(0.62)
Medicaid	38.02	41.35	52.08	66.04	10.25***	(0.72)	10.24***	(0.68)
Private								
Employer-sponsored	16.40	21.15	16.01	18.10	−2.97***	(0.56)	−2.87***	(0.51)
Directly-purchased	5.93	9.74	6.27	7.90	−1.77***	(0.30)	−1.72***	(0.30)
(Observations = 355,606)								
Above100% FPL								
Uninsured	33.54	21.45	26.61	12.06	−2.38***	(0.47)	−2.59***	(0.46)
Medicaid	15.02	16.41	22.13	32.44	8.73***	(0.42)	8.42***	(0.42)
Private								
Employer-sponsored	39.34	45.18	41.72	43.83	−3.65***	(0.53)	−3.13***	(0.46)
Directly-purchased	7.76	13.85	8.17	11.40	−2.80***	(0.36)	−2.77***	(0.35)
(Observations = 557,721)								

Notes: The sample used in this analysis excludes any states that implemented Medicaid expansion before 2014 or later than 2014. That is, we exclude 5 early expansion states: CA, DC, MA, MN, WA; and 6 late expansion states. Model adjusted for individuals' age, sex, race, level of education, citizenship status, marriage status, and employment status.

Table A2: Average Estimated Treatment Effect of 2014 Medicaid Expansion on Insurance Coverage Rates (For Income-to-Poverty Ratio above 400 Only)

	States %							
	Non-Expansion		Expansion		Unadjusted Difference-in-Differences (standard error)		Adjusted Difference-in-Differences (standard error)	
	Pre-ACA 2008-2013 (1)	Post-ACA 2015-2018 (2)	Pre-ACA 2008-2013 (3)	Post-ACA 2015-2018 (4)	(5)		(6)	
Panel A: RTI up to 30%								
**Income-to-poverty ratio > 400**								
Uninsured	11.87	8.37	9.12	4.97	−0.73**	(0.31)	−0.53	(0.30)
Medicaid	2.39	2.89	3.44	5.46	1.60***	(0.18)	1.76***	(0.18)
Private								
Employer-sponsored	75.85	77.60	81.01	81.95	−0.77	(0.42)	−0.94*	(0.39)
Directly-purchased	8.39	9.81	7.47	8.59	−0.70**	(0.25)	−0.67**	(0.25)
(Observations = 523,789)								
Panel B: RTI above 30%								
**Income-to-poverty ratio > 400**								
Uninsured	12.36	7.63	8.12	5.09	1.46	(1.20)	1.73	(1.20)
Medicaid	1.35	1.42	1.98	4.07	2.09***	(0.51)	1.96***	(0.52)
Employer-sponsored	68.61	70.54	75.13	74.18	−2.29	(2.29)	−0.81	(2.19)
Directly-purchased	15.53	19.08	11.81	13.30	−3.06	(1.76)	−3.17	(1.74)
(Observations = 23,022)								

Notes: The sample used in this analysis exclude any states that implement Medicaid expansion before 2014. That is, we exclude 5 early expansion states: CA, DC, MA, MN, WA; and 6 late expansion states. Column (5) presents the unadjusted DID estimates without any household-level controls. Column (6) presents the adjusted DID estimates, by controlling for respondents' age, sex, race, level of education, citizenship status, marriage status, and employment status. We controlled for both year and state fixed effect in columns (5) and (6). The standard errors clustered at CPUMA level are in the parentheses. $***$, $**$ and $*$ indicate significance at levels 1%, 5%, and 10%.

B Demographic Difference and Self-Selection

Low-income households are more likely to fall into the high RTI group. Therefore, we control the household income-to-poverty ratio in our benchmark regression (Table 3). In our robustness check, we first test whether different measures of household income influence the outcomes. In Table B1, control for annual household income instead of the income-to-poverty ratio. Our results in Table B1 are consistent with the findings in Table 3, though the estimated treatment effects on the outcome variables show slight variation, suggesting that using different measurements to control income levels does not significantly impact the treatment effects on outcome variables.

Table B1: Impact of Medicaid Expansion on Health Insurance Coverage: Using Annual Household Income Instead of Income-to-Poverty Ratio

	All Income Level				Income Above 100 FPL			
	Uninsured	Medicaid	Employer	Direct	Uninsured	Medicaid	Employer	Direct
	(1)	(2)	(3)	(4)	(5)	(6)	(7)	(8)
$HighRent \times Expanded \times Post$	0.003	0.016^{***}	-0.010^{**}	-0.010^{***}	0.007^{*}	0.016^{***}	-0.010^{*}	-0.015^{***}
	(0.004)	(0.004)	(0.005)	(0.003)	(0.004)	(0.004)	(0.006)	(0.003)
$Expanded \times Post$	-0.036^{***}	0.077^{***}	-0.026^{***}	-0.014^{***}	-0.032^{***}	0.072^{***}	-0.026^{***}	-0.013^{***}
	(0.003)	(0.003)	(0.003)	(0.002)	(0.003)	(0.003)	(0.003)	(0.002)
No. of Observations	1,969,099	1,969,099	1,969,099	1,969,099	1,490,705	1,490,705	1,490,705	1,490,705
Year FE	Yes	Yes	Yes	Yes	Yes	Yes	Yes	Yes
State FE	Yes	Yes	Yes	Yes	Yes	Yes	Yes	Yes

Notes: The sample used in this analysis excludes five early expansion states (CA, MA, MN, WA, DC), as well as states that expanded after 2015 but before 2020 (AK, IN, LA, ME, MT, VA). Columns (1) to (4) are estimated coefficients based on full samples. Columns (5) to (8) are estimated coefficients based on subsample with annual income above 100% FPL. Regressions control for respondents' age, sex, race, level of education, household income, citizenship status, marriage status, and employment status. The standard errors clustered at CPUMA level are in the parentheses. $***$, $**$ and $*$ indicate significance at levels 1%, 5%, and 10%.

Moreover, we compare the demographic information between the expansion states and non-expansion states and check whether there exist differences between these two types of states that would bias our findings. In Tables B2 and B3, we summarize their descriptive statistics. Specifically, Table B2 consists of the pre-ACA statistics and Table B3 depicts the entire sample statistics. Overall, these two tables indicate that there is little difference between the statistics of expansion states and non-expansion states, showing that the composition of a state is not significantly affected by its expansion status. These findings suggest that the effects of self-selection in expansion states caused by Medicaid expansion are limited.

Table B2: Pre-Expansion Descriptive Statistics (Expansion states vs. Non-expansion states)

Variables	Full Sample		Rent-to-income Ratio			
			Up to 30%		Above 30%	
	Expansion	Non-Expansion	Expansion	Non-Expansion	Expansion	Non-Expansion
Household Income (in FPL)						
0-100% FPL	25.58%	25.91%	12.35%	11.22%	38.72%	42.25%
101-400% FPL	74.42%	74.09%	87.65%	88.78%	61.28%	57.75%
Age	37.25	36.73	36.93	36.40	37.60	37.10
Unemployment Rate	10.07%	9.47%	8.19%	7.56 %	12.08%	11.60 %
Percent of Married Household	29.98%	32.65%	32.78%	36.86%	27.01%	27.97%
Female Respondent	54.47%	55.11%	52.40%	52.50%	56.67%	58.01%
Race:						
White	70.21%	65.94%	72.63%	69.09%	67.63%	62.43%
Black or African Native	18.08%	26.72%	16.07%	23.38%	20.22%	30.44%
Asian	2.52%	1.10%	2.33%	1.09%	2.72%	1.12%
Muti-racial	2.97%	2.40%	2.71%	2.33%	3.26%	2.48%
Other	6.21%	3.83%	6.26%	4.11%	6.17%	3.53%
Insurance Coverage:						
Medicaid	26.15%	18.89%	18.84%	13.48%	33.93%	24.91%
Employer-sponsored	42.49%	40.28%	52.74%	49.97%	31.59%	29.48%
Directly-purchased	6.94%	6.64%	6.49%	6.34%	7.42%	6.97%
Uninsured Rate	24.70%	32.30%	22.96%	29.01%	26.56%	35.96%
No. Of Observations	455,248	408,406	215,157	229,361	220,616	193,249

Notes: Mean of the variables in our sample are reported in this table and calculations are based on ACS 2010-18. Sample is restricted to those between 18 and 65, up to 400% FPL, and not covered by VA Health Care or Indian Health Service. Other racial groups include American Indian, Native Hawaiian, Pacific Islander and Alaska Native. All variables are binary except for age, household income and family size, which are continuous. Calculations account for ACS sample weights.

Table B3: Descriptive Statistics (Expansion states vs. Non-expansion states)

Variables	Full Sample		Rent-to-income Ratio			
			Up to 30%		Above 30%	
	Expansion	Non-Expansion	Expansion	Non-Expansion	Expansion	Non-Expansion
Household Income (in FPL)						
0-100% FPL	24.21%	24.39%	12.26%	10.96%	37.68%	40.35%
101-400% FPL	75.79%	75.61%	87.74%	89.04%	62.32%	59.65%
Age	37.24	36.84	36.77	36.44	37.77	37.33
Unemployment Rate	7.80%	7.25%	6.34%	5.86 %	9.44%	8.91 %
Percent of Married Household	29.38%	32.28%	31.80%	36.02%	26.65%	27.84%
Female Respondent	54.32%	54.95%	52.35%	52.47%	56.54%	57.90%
Race:						
White	70.01%	66.28%	72.49%	69.06%	67.22%	62.98%
Black or African Native	17.62%	25.92%	15.71%	23.01%	19.78%	29.37%
Asian	2.71%	1.23%	2.41%	1.18%	3.06%	1.28%
Muti-racial	2.88%	2.64%	2.64%	2.57%	2.64%	2.72%
Other	6.41%	3.93%	6.39%	4.18%	6.44%	3.64%
Insurance Coverage:						
Medicaid	30.42%	19.19%	22.76%	14.01%	39.05%	25.34%
Employer-sponsored	44.24%	43.38%	54.11%	52.31%	33.11%	32.76%
Directly-purchased	8.21%	8.94%	7.70%	8.32%	8.78%	9.67%
Uninsured Rate	17.87%	26.99%	16.88%	24.54%	18.97%	29.90%
No. Of Observations	1,028,690	940,409	545,086	510,686	483,604	429,723

Notes: Mean of the variables in our sample are reported in this table and calculations are based on ACS 2010-18. Sample is restricted to those between 18 and 65, up to 400% FPL, and not covered by VA Health Care or Indian Health Service. Other racial groups include American Indian, Native Hawaiian, Pacific Islander and Alaska Native. All variables are binary except for age, household income and family size, which are continuous. Calculations account for ACS sample weights.

C Results on Later Expansion States

Table C1: Status of State Action on the Medicaid Expansion Decision

**Expansion Status**	States
Early expansion states<br>(Expanded before 2014)	California, District of Columbia, Massachusetts, Minnesota, Washington
Expansion states<br>(Expanded in 2014)<br>(Exclude five early expansion states)	Arizona, Arkansas, Colorado, Connecticut, Delaware, Hawaii, Illinois, Iowa, Kentucky, Maryland, Michigan, Nevada, New Hampshire, New Jersey, New Mexico, New York, North Dakota, Ohio, Oregon, Pennsylvania, Rhode Island, Vermont, West Virginia
Non-expansion states<br>(Did not expand between 2015 and 2018)	Alabama, Florida, Georgia, Idaho, Kansas, Mississippi, Missouri, Nebraska, North Carolina, Oklahoma, South Carolina, South Dakota, Tennessee, Texas, Utah, Wisconsin, Wyoming
Late expansion states<br>(Expanded between 2015 and 2018)	Alaska, Indiana, Louisiana, Maine, Montana, Virginia

Sources: Kaiser Family Foundation

Table C2: Average Estimated Treatment Effect of 2015 and 2016 Medicaid Expansion on Insurance Coverage Rates

	Expand in 2015				Expand in 2016			
	Unadjusted Difference-in-Differences (standard error) (1)		Adjusted Difference-in-Differences (standard error) (2)		Unadjusted Difference-in-Differences (standard error) (3)		Adjusted Difference-in-Differences (standard error) (4)	
Panel A: Total Sample								
Uninsured	-4.99^{***}	(0.61)	-4.88^{***}	(0.56)	-10.92^{***}	(0.80)	-11.36^{***}	(0.84)
Medicaid	5.43^{***}	(0.60)	5.37^{***}	(0.50)	14.87^{***}	(1.27)	13.93^{***}	(0.86)
Private								
Employer-sponsored	0.44	(1.00)	0.47	(0.74)	−2.14	(1.31)	−0.70	(0.96)
Directly-purchased	−0.53	(0.61)	−0.50	(0.61)	-1.13^{*}	(0.64)	-1.13^{*}	(0.67)
(Obs = 1,005,089 in 2015)								
(Obs = 991,318 in 2016)								
Panel B: Different Income Group								
**non-rent-burdened group**: RTI up to 30%								
Uninsured	-4.54^{***}	(0.57)	-4.24^{***}	(0.52)	-10.58^{***}	(0.77)	-10.74^{***}	(0.78)
Medicaid	3.92^{***}	(0.79)	3.75^{***}	(0.69)	10.92^{***}	(1.18)	10.42^{***}	(0.94)
Private								
Employer-sponsored	0.79	(1.33)	1.17	(1.14)	0.85	(1.07)	1.67	(1.09)
Directly-purchased	−0.59	(0.59)	−0.59	(0.59)	−0.47	(0.81)	−0.54	(0.82)
(Obs = 548,470 in 2015)								
(Obs = 537,648 in 2016)								
**rent-burdened group**: RTI above 30%								
Uninsured	-5.65^{***}	(1.11)	-6.00^{***}	(1.07)	-11.42^{***}	(1.21)	-11.92^{***}	(1.31)
Medicaid	8.55^{***}	(0.95)	7.92^{***}	(0.90)	18.11^{***}	(1.34)	17.46^{***}	(1.15)
Private								
Employer-sponsored	−1.34	(1.08)	−0.42	(0.92)	-4.21^{***}	(1.35)	-3.10^{**}	(1.20)
Directly-purchased	−0.28	(1.00)	−0.16	(1.00)	-1.91^{**}	(0.90)	-1.74^{*}	(0.97)
(Obs = 456,619 in 2015)								
(Obs = 453,670 in 2016)								

Notes: The sample used in this analysis exclude any states that implement Medicaid expansion before 2014 or later than 2014. That is, we exclude 5 early expansion states: CA, DC, MA, MN, WA; and 6 late expansion states. Adjusted DID estimates control for respondents' age, sex, race, level of education, income-to-poverty ratio, citizenship status, marriage status, and employment status. We controlled for both year and state fixed effect in columns (5) and (6). The standard errors clustered at CPUMA level are in the parentheses. $***$, $**$ and $*$ indicate significance at levels 1%, 5%, and 10%.

Figure C1: Estimated Treatment Effect Across Year (Expansion in 2015)

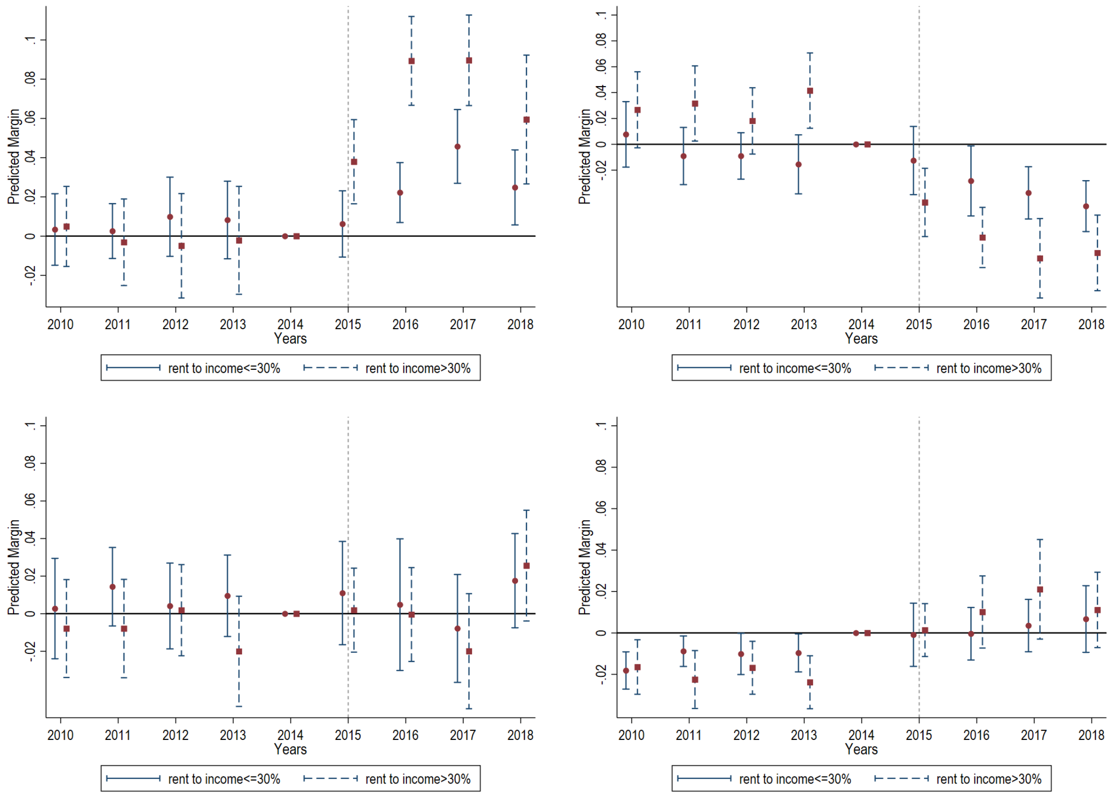

Notes: The figure is based on a sample that excludes any early or late expansion states. The estimation is based on states that adopted Medicaid expansion in 2015 and those that did not participate. The red dots (or red squares) is the estimated average treatment effect on the non-rent-burdened group (or rent-burdened group). The solid line (or dash line) is the 95% confidence interval for estimated average treatment effect on the non-rent-burdened group (or rent-burdened group). Upper Left: Medicaid coverage rate. Upper Right: Uninsured rate. Lower Left: employer-sponsored rate. Lower Right: directly-purchased rate. For state implement Medicaid expansion in 2015.

Figure C2: Estimated Treatment Effect Across Year (Expansion in 2016)

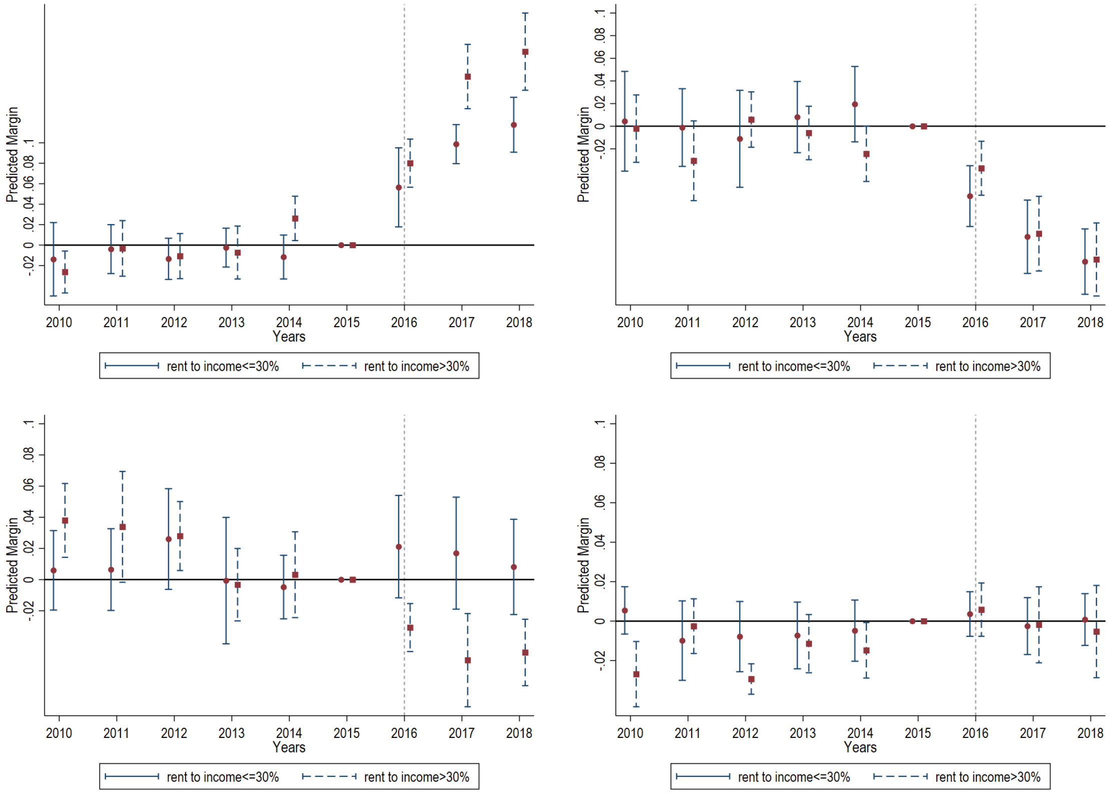

Notes: The figure is based on a sample that excludes any early or late expansion states. The estimation is based on states that adopted Medicaid expansion in 2016 and those that did not participate. The red dots (or red squares) is the estimated average treatment effect on the non-rent-burdened group (or rent-burdened group). The solid line (or dash line) is the 95% confidence interval for estimated average treatment effect on the non-rent-burdened group (or rent-burdened group). Upper Left: Medicaid coverage rate. Upper Right: Uninsured rate. Lower Left: employer-sponsored rate. Lower Right: directly-purchased rate. For state implement Medicaid expansion in 2016.

D Parallel Testing and Complete Triple Difference Results

Table D1: Pre-Expansion Parallel DD

	Unadjusted DID	Adjusted DID with Demographic Controls		
	Full Sample	Full Sample	non-Rent-Burdened	Rent-Burdened
	(1)	(2)	(3)	(4)
Uninsured	0.195	0.155	−0.059	0.352
	(0.145)	(0.143)	(0.185)	(0.188)
Medicaid	−0.035	−0.079	0.114	−0.248
	(0.130)	(0.123)	(0.135)	(0.172)
Employer-Sponsored	−0.134	−0.072	0.037	−0.188
	(0.160)	(0.142)	(0.188)	(0.181)
Directly-Purchased	−0.195**	−0.185**	−0.174*	−0.197*
	(0.077)	(0.077)	(0.104)	(0.100)
No. Observations	863,654	863,654	449,789	413,865

Notes: The sample used in this analysis exclude any states that implement Medicaid expansion before 2014. That is, we exclude 5 early expansion states: CA, DC, MA, MN, WA; and 6 late expansion states. Column (1) presents the unadjusted DID estimates (in percentage points) without any household-level controls. Columns (2) to (4) presents the adjusted DID estimates (in percentage points), by controlling for respondents' age, sex, race, level of education, income-to-poverty ratio, citizenship status, marriage status, and employment status. We controlled for both year and state fixed effect for all columns. The standard errors clustered at CPUMA level are in the parentheses. ∗∗∗, ∗∗ and ∗ indicate significance at levels 1%, 5%, and 10%.

Table D2: Triple Difference Regression Dynamic Results

	All Income Level				Income Above 100% FPL			
	Uninsured	Medicaid	Employer	Direct	Uninsured	Medicaid	Employer	Direct
	(1)	(2)	(3)	(4)	(5)	(6)	(7)	(8)
$HighRent \times Expanded \times D_{2010}$	-0.010	0.006	0.010	-0.001	-0.013	0.005	0.014	-0.000
	(0.007)	(0.006)	(0.008)	(0.004)	(0.008)	(0.007)	(0.009)	(0.005)
$HighRent \times Expanded \times D_{2011}$	-0.004	0.010	-0.002	0.003	0.004	0.008	-0.007	0.005
	(0.009)	(0.007)	(0.008)	(0.004)	(0.010)	(0.007)	(0.010)	(0.005)
$HighRent \times Expanded \times D_{2012}$	0.005	-0.002	0.003	-0.003	0.003	0.002	0.001	-0.000
	(0.008)	(0.006)	(0.009)	(0.004)	(0.009)	(0.007)	(0.010)	(0.005)
$HighRent \times Expanded \times D_{2014}$	-0.003	0.014**	0.001	-0.007*	0.002	0.020***	-0.008	-0.010*
	(0.008)	(0.006)	(0.008)	(0.004)	(0.009)	(0.007)	(0.010)	(0.005)
$HighRent \times Expanded \times D_{2015}$	0.005	0.019***	-0.009	-0.011**	0.013	0.020***	-0.010	-0.018***
	(0.008)	(0.007)	(0.008)	(0.005)	(0.009)	(0.007)	(0.010)	(0.006)
$HighRent \times Expanded \times D_{2016}$	-0.000	0.023***	-0.011	-0.012**	0.007	0.024***	-0.013	-0.016***
	(0.007)	(0.007)	(0.008)	(0.005)	(0.008)	(0.007)	(0.010)	(0.006)
$HighRent \times Expanded \times D_{2017}$	-0.004	0.018***	-0.002	-0.007	-0.000	0.019***	-0.008	-0.008
	(0.008)	(0.007)	(0.008)	(0.005)	(0.008)	(0.007)	(0.010)	(0.006)
$HighRent \times Expanded \times D_{2018}$	0.003	0.014*	-0.001	-0.010**	0.004	0.015**	0.001	-0.014**
	(0.008)	(0.007)	(0.009)	(0.005)	(0.008)	(0.008)	(0.010)	(0.006)
$Expanded \times D_{2010}$	-0.001	-0.002	-0.000	0.006*	-0.001	-0.002	0.001	0.006*
	(0.006)	(0.004)	(0.006)	(0.003)	(0.006)	(0.004)	(0.006)	(0.003)
$Expanded \times D_{2011}$	-0.002	-0.003	0.002	0.002	-0.004	-0.000	0.003	0.001
	(0.006)	(0.004)	(0.006)	(0.003)	(0.006)	(0.004)	(0.006)	(0.003)
$Expanded \times D_{2012}$	-0.009*	0.001	0.004	0.006*	-0.011*	0.001	0.005	0.006*
	(0.005)	(0.004)	(0.006)	(0.003)	(0.006)	(0.004)	(0.006)	(0.003)
$Expanded \times D_{2014}$	-0.024***	0.032***	-0.007	-0.003	-0.024***	0.028***	-0.004	-0.002
	(0.005)	(0.004)	(0.006)	(0.003)	(0.005)	(0.004)	(0.006)	(0.003)
$Expanded \times D_{2015}$	-0.035***	0.062***	-0.013**	-0.014***	-0.031***	0.058***	-0.013**	-0.013***
	(0.005)	(0.005)	(0.006)	(0.003)	(0.006)	(0.005)	(0.006)	(0.004)
$Expanded \times D_{2016}$	-0.036***	0.074***	-0.020***	-0.013***	-0.033***	0.070***	-0.022***	-0.012***
	(0.005)	(0.005)	(0.006)	(0.003)	(0.005)	(0.005)	(0.006)	(0.003)
$Expanded \times D_{2017}$	-0.041***	0.080***	-0.031***	-0.010***	-0.040***	0.078***	-0.029***	-0.010***
	(0.006)	(0.005)	(0.006)	(0.003)	(0.006)	(0.005)	(0.006)	(0.003)
$Expanded \times D_{2018}$	-0.045***	0.081***	-0.027***	-0.006	-0.043***	0.077***	-0.027***	-0.003
	(0.005)	(0.005)	(0.006)	(0.004)	(0.005)	(0.005)	(0.006)	(0.004)
No. of Observations	1,969,099	1,969,099	1,969,099	1,969,099	1,490,705	1,490,705	1,490,705	1,490,705
Year FE	Yes	Yes	Yes	Yes	Yes	Yes	Yes	Yes
State FE	Yes	Yes	Yes	Yes	Yes	Yes	Yes	Yes

Notes: The sample used in this analysis excludes five early expansion states (CA, MA, MN, WA, DC), as well as states that expanded after 2015 but before 2020 (AK, IN, LA, ME, MT, VA). Columns (1) to (4) are estimated coefficients based on full samples. Columns (5) to (8) are estimated coefficients based on the subsample with annual income above 100 FPL. Other controls in equation (2) are included but not reported. Complete DDD results can be found in Table D3 in the Appendix. The standard errors clustered at CPUMA level are in the parentheses. $***$, $**$ and $*$ indicate significance at levels 1%, 5%, and 10%.

Table D3: Complete Triple Difference Regression Results

	All Income Level				Income Above 100% FPL			
	Uninsured (1)	Medicaid (2)	Employer (3)	Direct (4)	Uninsured (5)	Medicaid (6)	Employer (7)	Direct (8)
$HighRent \times Expanded \times Post$	0.002	0.015***	−0.008*	−0.010**	0.007	0.015***	−0.008	−0.015**
	(0.004)	(0.004)	(0.005)	(0.003)	(0.004)	(0.004)	(0.005)	(0.003)
$Expanded \times Post$	−0.037**	0.075***	−0.024**	−0.014**	−0.033***	0.071***	−0.025**	−0.013**
	(0.003)	(0.003)	(0.003)	(0.002)	(0.003)	(0.003)	(0.003)	(0.002)
$HighRent \times Post$	−0.029***	0.014***	0.007**	0.012***	−0.036***	0.012***	0.008*	0.021***
	(0.003)	(0.002)	(0.003)	(0.002)	(0.003)	(0.003)	(0.004)	(0.002)
$HighRent \times Expanded$	−0.028***	0.046***	−0.020***	0.035*	−0.009***	0.030***	−0.021***	0.005**
	(0.004)	(0.004)	(0.004)	(0.002)	(0.004)	(0.003)	(0.004)	(0.002)
$Post$	−0.086***	0.037***	0.028***	0.027***	−0.079***	0.035***	0.021***	0.027***
	(0.003)	(0.003)	(0.003)	(0.002)	(0.003)	(0.003)	(0.003)	(0.002)
$Expanded$	−0.019*	0.081***	−0.019	−0.034***	−0.004	0.056***	−0.014	−0.032***
	(0.011)	(0.016)	(0.015)	(0.008)	(0.010)	(0.010)	(0.012)	(0.007)
$HighRent$	0.027***	−0.042***	−0.009***	0.010***	0.021***	−0.049***	−0.003	0.012***
	(0.003)	(0.003)	(0.003)	(0.001)	(0.003)	(0.002)	(0.003)	(0.002)
$Expanded \times Year\,2014$	−0.021***	0.033***	−0.009*	−0.007***	−0.020***	0.029***	−0.008	−0.005***
	(0.004)	(0.004)	(0.005)	(0.003)	(0.004)	(0.004)	(0.005)	(0.003)
$HighRent \times Year\,2014$	−0.015***	0.012***	−0.008*	0.008***	−0.019***	0.006	−0.002	0.013***
	(0.005)	(0.003)	(0.004)	(0.002)	(0.005)	(0.004)	(0.005)	(0.003)
$HighRent \times Expanded \times Year\,2014$	−0.001	0.010*	−0.000	−0.007**	0.003	0.015**	−0.008	−0.011***
	(0.006)	(0.005)	(0.006)	(0.004)	(0.007)	(0.006)	(0.007)	(0.004)
No. of Observations	1,969,099	1,969,099	1,969,099	1,969,099	1,490,705	1,490,705	1,490,705	1,490,705
Year FE	Yes	Yes	Yes	Yes	Yes	Yes	Yes	Yes
State FE	Yes	Yes	Yes	Yes	Yes	Yes	Yes	Yes

Notes: The sample used in this analysis exclude any states that implement Medicaid expansion before 2014 or later than 2014. That is, we exclude 5 early expansion states: CA, DC, MA, MN, WA; and 6 late expansion states. D_i is a dummy variable that equals 1 if in year i. Columns (1) to (4) are estimated coefficients based on full samples. Columns (5) to (8) are estimated coefficients based on subsample with annual income above 100 federal poverty line. Regressions control for respondents' age, sex, race, level of education, income-to-poverty ratio, citizenship status, marriage status, and employment status. The standard errors clustered at CPUMA level are in the parentheses. $***$, $**$ and $*$ indicate significance at levels 1%, 5%, and 10%.

E Tables for Major Robustness Check

E.1 Early ACA provision and age group

Table E1: Effect of Medicaid Expansion on Health Insurance Coverage by Age Group (Above and Up to 26 Years)

	All Income Level				Income Above 100 FPL			
	Uninsured	Medicaid	Employer	Direct	Uninsured	Medicaid	Employer	Direct
	(1)	(2)	(3)	(4)	(5)	(6)	(7)	(8)
Panel A: Age above 26								
HighRent × *Expanded* × *Post*	0.005	0.020***	−0.013***	−0.012***	0.009*	0.017***	−0.011*	−0.017***
	(0.004)	(0.004)	(0.005)	(0.003)	(0.005)	(0.005)	(0.006)	(0.003)
Expanded × *Post*	−0.038***	0.075***	−0.020***	−0.016***	−0.033***	0.071***	−0.021***	−0.015***
	(0.003)	(0.004)	(0.003)	(0.002)	(0.003)	(0.003)	(0.004)	(0.002)
No. of Observations	1,414,438	1,414,438	1,414,438	1,414,438	1,099,212	1,099,212	1,099,212	1,099,212
Panel B: Age up to 26								
HighRent × *Expanded* × *Post*	−0.003	0.008	−0.000	−0.006	−0.000	0.013*	−0.003	−0.010*
	(0.007)	(0.006)	(0.008)	(0.005)	(0.008)	(0.007)	(0.010)	(0.006)
Expanded × *Post*	−0.036***	0.071***	−0.026***	−0.010**	−0.034***	0.070***	−0.029***	−0.007**
	(0.006)	(0.005)	(0.006)	(0.003)	(0.006)	(0.005)	(0.006)	(0.004)
No. of Observations	554,661	554,661	554,661	554,661	391,493	391,493	391,493	391,493
Year FE	Yes	Yes	Yes	Yes	Yes	Yes	Yes	Yes
State FE	Yes	Yes	Yes	Yes	Yes	Yes	Yes	Yes

Notes: The sample used in this analysis excludes five early expansion states (CA, MA, MN, WA, DC), as well as states that expanded after 2015 but before 2020 (AK, IN, LA, ME, MT, VA). Columns (1) to (4) are estimated coefficients based on full samples. Columns (5) to (8) are estimated coefficients based on subsample with annual income above 100% FPL. Regressions control for respondents' age, sex, race, level of education, household income, citizenship status, marriage status, and employment status. The standard errors clustered at CPUMA level are in the parentheses. ∗∗∗, ∗∗ and ∗ indicate significance at levels 1%, 5%, and 10%.

Table E2: Effect of Medicaid Expansion on Health Insurance Coverage: Excluding the Year 2010

	All Income Level				Income Above 100 FPL			
	Uninsured	Medicaid	Employer	Direct	Uninsured	Medicaid	Employer	Direct
	(1)	(2)	(3)	(4)	(5)	(6)	(7)	(8)
$HighRent \times Expanded \times Post$	-0.002	0.017^{***}	-0.006	-0.010^{***}	-0.000	0.019^{***}	-0.005	-0.016^{***}
	(0.004)	(0.004)	(0.004)	(0.002)	(0.004)	(0.004)	(0.005)	(0.003)
$Expanded \times Post$	-0.042^{***}	0.084^{***}	-0.027^{***}	-0.013^{***}	-0.039^{***}	0.081^{***}	-0.027^{***}	-0.011^{***}
	(0.004)	(0.003)	(0.003)	(0.002)	(0.004)	(0.003)	(0.004)	(0.002)
No. of Observations	2,220,285	2,220,285	2,220,285	2,220,285	1,702,622	1,702,622	1,702,622	1,702,622
Year FE	Yes	Yes	Yes	Yes	Yes	Yes	Yes	Yes
State FE	Yes	Yes	Yes	Yes	Yes	Yes	Yes	Yes

Notes: The sample used in this analysis excludes five early expansion states (CA, MA, MN, WA, DC), as well as states that expanded after 2015 but before 2020 (AK, IN, LA, ME, MT, VA). Columns (1) to (4) are estimated coefficients based on full samples. Columns (5) to (8) are estimated coefficients based on subsample with annual income above 100% FPL. Regressions control for respondents' age, sex, race, level of education, household income, citizenship status, marriage status, and employment status. The standard errors clustered at state level are in the parentheses. $***$, $**$ and $*$ indicate significance at levels 1%, 5%, and 10%.

Table E3: Effect of Medicaid Expansion on Health Insurance Coverage: Excluding the Year 2010 and 2011

	All Income Level				Income Above 100 FPL			
	Uninsured	Medicaid	Employer	Direct	Uninsured	Medicaid	Employer	Direct
	(1)	(2)	(3)	(4)	(5)	(6)	(7)	(8)
$HighRent \times Expanded \times Post$	-0.004	0.020^{***}	-0.007	-0.009^{***}	0.000	0.022^{***}	-0.008	-0.014^{***}
	(0.004)	(0.004)	(0.005)	(0.003)	(0.005)	(0.004)	(0.006)	(0.003)
$Expanded \times Post$	-0.041^{***}	0.083^{***}	-0.026^{***}	-0.013^{***}	-0.039^{***}	0.080^{***}	-0.026^{***}	-0.012^{***}
	(0.004)	(0.004)	(0.004)	(0.002)	(0.004)	(0.003)	(0.004)	(0.002)
No. of Observations	1,949,693	1,949,693	1,949,693	1,949,693	1,500,912	1,500,912	1,500,912	1,500,912
Year FE	Yes	Yes	Yes	Yes	Yes	Yes	Yes	Yes
State FE	Yes	Yes	Yes	Yes	Yes	Yes	Yes	Yes

Notes: The sample used in this analysis excludes states that expanded after 2015 but before 2020 (AK, IN, LA, ME, MT, VA). Columns (1) to (4) are estimated coefficients based on full samples. Columns (5) to (8) are estimated coefficients based on subsample with annual income above 100% FPL. Regressions control for respondents' age, sex, race, level of education, household income, citizenship status, marriage status, and employment status. The standard errors clustered at state level are in the parentheses. $***$, $**$ and $*$ indicate significance at levels 1%, 5%, and 10%.

E.2 Different Rent-to-Income Threshold

Table E4: Effect of Medicaid Expansion on Health Insurance Coverage with Varying Rent-to-Income Thresholds

	All Income Level				Income Above 100% FPL			
	Uninsured	Medicaid	Employer	Direct	Uninsured	Medicaid	Employer	Direct
	(1)	(2)	(3)	(4)	(5)	(6)	(7)	(8)
Panel A: RTI threshold = 40%								
$HighRent \times Expanded \times Post$	0.003	0.015***	−0.006	−0.011***	0.007	0.023***	−0.010	−0.019***
	(0.004)	(0.005)	(0.005)	(0.003)	(0.005)	(0.005)	(0.006)	(0.004)
$Expanded \times Post$	−0.037***	0.078***	−0.026***	−0.016***	−0.031***	0.072***	−0.025***	−0.015***
	(0.003)	(0.003)	(0.003)	(0.002)	(0.003)	(0.003)	(0.003)	(0.002)
Panel B: RTI threshold = 50%								
$HighRent \times Expanded \times Post$	0.001	0.013***	−0.001	−0.013***	0.009	0.024***	−0.005	−0.027***
	(0.005)	(0.005)	(0.006)	(0.003)	(0.008)	(0.007)	(0.009)	(0.006)
$Expanded \times Post$	−0.036***	0.080***	−0.027***	−0.016***	−0.031***	0.074***	−0.027***	−0.016***
	(0.003)	(0.003)	(0.003)	(0.002)	(0.003)	(0.003)	(0.003)	(0.002)
Panel C: RTI threshold = 60%								
$HighRent \times Expanded \times Post$	0.005	0.011	−0.000	−0.013***	0.017	0.023**	−0.002	−0.028***
	(0.006)	(0.007)	(0.006)	(0.004)	(0.011)	(0.010)	(0.012)	(0.007)
$Expanded \times Post$	−0.036***	0.081***	−0.027***	−0.017***	−0.031***	0.075***	−0.027***	−0.017***
	(0.003)	(0.003)	(0.003)	(0.002)	(0.003)	(0.003)	(0.003)	(0.002)
Panel D: Use Rent-to-Income as Continuous Variable								
$RTI \times Expanded \times Post$	0.005	0.030***	−0.007	−0.027***	0.018	0.045***	−0.008	−0.057***
	(0.010)	(0.010)	(0.010)	(0.006)	(0.014)	(0.013)	(0.017)	(0.010)
$Expanded \times Post$	−0.033***	0.064***	−0.023***	−0.007***	−0.031***	0.056***	−0.023***	0.000
	(0.004)	(0.004)	(0.004)	(0.002)	(0.004)	(0.005)	(0.006)	(0.003)
No. of Observations	1,969,099	1,969,099	1,969,099	1,969,099	1,490,705	1,490,705	1,490,705	1,490,705
Year FE	Yes	Yes	Yes	Yes	Yes	Yes	Yes	Yes
State FE	Yes	Yes	Yes	Yes	Yes	Yes	Yes	Yes

Notes: The sample used in this analysis exclude any states that implement Medicaid expansion before 2014 or later than 2014. That is, we exclude 5 early expansion states: CA, DC, MA, MN, WA; and 6 late expansion states. Columns (1) to (4) are estimated coefficients based on full samples. Columns (5) to (8) are estimated coefficients based on subsample with annual income above 100 federal poverty line. Regressions control for respondents' age, sex, race, level of education, income-to-poverty ratio, citizenship status, marriage status, and employment status. The standard errors clustered at CPUMA level are in the parentheses. $***$, $**$ and $*$ indicate significance at levels 1%, 5%, and 10%.

Table E5: Tripple difference results within rent-burdened household ($RTI > 30\%$)

	All Income Level				Income Above 100% FPL			
	Uninsured	Medicaid	Employer	Direct	Uninsured	Medicaid	Employer	Direct
	(1)	(2)	(3)	(4)	(5)	(6)	(7)	(8)
Panel A: RTI threshold = 40%								
$HighRent \times Expanded \times Post$	0.003	0.007	−0.000	−0.007*	0.002	0.021***	−0.006	−0.012***
	(0.006)	(0.006)	(0.006)	(0.004)	(0.007)	(0.006)	(0.008)	(0.004)
$Expanded \times Post$	−0.036***	0.086***	−0.032***	−0.020***	−0.026***	0.074***	−0.029***	−0.022***
	(0.005)	(0.005)	(0.005)	(0.003)	(0.005)	(0.005)	(0.006)	(0.004)
No. of Observations	913,327	913,327	913,327	913,327	557,721	557,721	557,721	557,721
Panel B: RTI threshold = 50%								
$HighRent \times Expanded \times Post$	−0.011	0.012	0.003	−0.003	−0.003	0.006	−0.007	−0.002
	(0.008)	(0.008)	(0.009)	(0.006)	(0.012)	(0.011)	(0.013)	(0.009)
$Expanded \times Post$	−0.001	0.002	−0.002	0.001	−0.014	0.003	0.009	0.008
	(0.006)	(0.005)	(0.007)	(0.004)	(0.009)	(0.007)	(0.010)	(0.007)
No. of Observations	913,327	913,327	913,327	913,327	557,721	557,721	557,721	557,721
Panel C: RTI threshold = 60%								
$HighRent \times Expanded \times Post$	−0.001	0.013	−0.005	0.000	0.005	0.015	−0.004	−0.003
	(0.010)	(0.010)	(0.010)	(0.006)	(0.017)	(0.014)	(0.018)	(0.013)
$Expanded \times Post$	−0.002	0.000	−0.005	−0.003	−0.016	−0.007	−0.002	0.011
	(0.008)	(0.007)	(0.008)	(0.005)	(0.013)	(0.008)	(0.014)	(0.010)
No. of Observations	913,327	913,327	913,327	913,327	557,721	557,721	557,721	557,721
Year FE	Yes	Yes	Yes	Yes	Yes	Yes	Yes	Yes
State FE	Yes	Yes	Yes	Yes	Yes	Yes	Yes	Yes

Notes: The regression are based on rent-burdened household, i.e., those with rent-to-income ratio above 30%. The sample used in this analysis exclude any states that implement Medicaid expansion before 2014 or later than 2014. That is, we exclude 5 early expansion states: CA, DC, MA, MN, WA; and 6 late expansion states. Columns (1) to (4) are estimated coefficients based on full samples. Columns (5) to (8) are estimated coefficients based on subsample with annual income above 100 federal poverty line. Regressions control for respondents' age, sex, race, level of education, income-to-poverty ratio, citizenship status, marriage status, and employment status. The standard errors clustered at CPUMA level are in the parentheses. $***$, $**$ and $*$ indicate significance at levels 1%, 5%, and 10%.

E.3 State-level Policies

Table E6: Impact of State-level Policies on Health Insurance Coverage under Medicaid Expansion by Rent Burden and Income Level

	All Income Level				Income Above 100% FPL			
	Uninsured	Medicaid	Employer	Direct	Uninsured	Medicaid	Employer	Direct
	(1)	(2)	(3)	(4)	(5)	(6)	(7)	(8)
Panel A: Add State-Year FE								
HighRent × *Expanded* × *Post*	-0.003	0.019^{***}	-0.008^{*}	-0.008^{***}	-0.001	0.019^{***}	-0.007	-0.012^{***}
	(0.004)	(0.004)	(0.004)	(0.002)	(0.004)	(0.004)	(0.005)	(0.003)
Expanded × *Post*	-0.169^{***}	0.183^{***}	0.042	-0.033^{*}	-0.152^{***}	0.125^{***}	0.082	-0.025^{*}
	(0.027)	(0.023)	(0.057)	(0.020)	(0.054)	(0.014)	(0.070)	(0.013)
No. of Observations	1,969,099	1,969,099	1,969,099	1,969,099	1,490,705	1,490,705	1,490,705	1,490,705
Year FE	Yes	Yes	Yes	Yes	Yes	Yes	Yes	Yes
State FE	Yes	Yes	Yes	Yes	Yes	Yes	Yes	Yes
State-Year FE	Yes	Yes	Yes	Yes	Yes	Yes	Yes	Yes
Panel B: Drop Waiver								
HighRent × *Expanded* × *Post*	-0.003	0.018^{***}	-0.006	-0.009^{***}	-0.001	0.018^{***}	-0.004	-0.013^{***}
	(0.004)	(0.004)	(0.005)	(0.002)	(0.004)	(0.004)	(0.005)	(0.003)
Expanded × *Post*	-0.168^{***}	0.183^{***}	0.042	-0.033^{*}	-0.152^{***}	0.125^{***}	0.081	-0.025^{*}
	(0.027)	(0.023)	(0.057)	(0.020)	(0.054)	(0.014)	(0.070)	(0.013)
No. of Observations	1,845,733	1,845,733	1,845,733	1,845,733	1,403,604	1,403,604	1,403,604	1,403,604
Year FE	Yes	Yes	Yes	Yes	Yes	Yes	Yes	Yes
State FE	Yes	Yes	Yes	Yes	Yes	Yes	Yes	Yes
Panel C: Within State-run Only								
HighRent × *Expanded* × *Post*	-0.022^{***}	0.024^{***}	0.008^{*}	-0.001	-0.027^{***}	0.022^{***}	0.012^{**}	0.001
	(0.006)	(0.006)	(0.005)	(0.003)	(0.006)	(0.006)	(0.005)	(0.004)
Expanded × *Post*	-0.163^{***}	0.152^{***}	0.023	0.015^{***}	-0.153^{***}	0.140^{***}	0.026	0.013^{**}
	(0.010)	(0.021)	(0.021)	(0.006)	(0.011)	(0.019)	(0.022)	(0.006)
No. of Observations	331,082	331,082	331,082	331,082	261,367	261,367	261,367	261,367
Year FE	Yes	Yes	Yes	Yes	Yes	Yes	Yes	Yes
State FE	Yes	Yes	Yes	Yes	Yes	Yes	Yes	Yes
Panel D: Drop State-run								
HighRent × *Expanded* × *Post*	-0.009^{**}	0.025^{***}	-0.012^{**}	-0.007^{***}	-0.006	0.024^{***}	-0.012^{*}	-0.010^{***}
	(0.004)	(0.004)	(0.005)	(0.003)	(0.005)	(0.004)	(0.006)	(0.003)
Expanded × *Post*	-0.165^{***}	0.180^{***}	0.044	-0.034^{*}	-0.151^{***}	0.124^{***}	0.083	-0.025^{*}
	(0.027)	(0.023)	(0.057)	(0.020)	(0.055)	(0.014)	(0.070)	(0.013)
No. of Observations	1,638,017	1,638,017	1,638,017	1,638,017	1,229,338	1,229,338	1,229,338	1,229,338
Year FE	Yes	Yes	Yes	Yes	Yes	Yes	Yes	Yes
State FE	Yes	Yes	Yes	Yes	Yes	Yes	Yes	Yes

Notes: The sample used in this analysis exclude any states that implement Medicaid expansion before 2014 or later than 2014. That is, we exclude 5 early expansion states: CA, DC, MA, MN, WA; and 6 late expansion states. Columns (1) to (4) are estimated coefficients based on full samples. Columns (5) to (8) are estimated coefficients based on subsample with annual income above 100 federal poverty line. Regressions control for respondents' age, sex, race, level of education, income-to-poverty ratio, citizenship status, marriage status, and employment status. The standard errors clustered at CPUMA level are in the parentheses. $***$, $**$ and $*$ indicate significance at levels 1%, 5%, and 10%.

E.4 Childcare Cost and Housing Related Expenses

Table E7: Impact of Number of Children on Health Insurance Coverage by Rent Burden and Income Level

	All Income Level				Income Above 100 FPL			
	Uninsured	Medicaid	Employer	Direct	Uninsured	Medicaid	Employer	Direct
	(1)	(2)	(3)	(4)	(5)	(6)	(7)	(8)
$HighRent \times Expanded \times Post$	0.002	0.015***	−0.008*	−0.010***	0.006	0.015***	−0.007	−0.015***
	(0.004)	(0.004)	(0.004)	(0.003)	(0.004)	(0.004)	(0.005)	(0.003)
$Expanded \times Post$	−0.037***	0.075***	−0.024***	−0.014***	−0.033***	0.071***	−0.025***	−0.013***
	(0.003)	(0.003)	(0.003)	(0.002)	(0.003)	(0.003)	(0.003)	(0.002)
Number of children	−0.015***	0.042***	−0.006***	−0.014***	−0.017***	0.030***	0.009***	−0.012***
	(0.001)	(0.001)	(0.001)	(0.000)	(0.001)	(0.001)	(0.001)	(0.000)
No. of Observations	1,969,099	1,969,099	1,969,099	1,969,099	1,490,705	1,490,705	1,490,705	1,490,705
Year FE	Yes	Yes	Yes	Yes	Yes	Yes	Yes	Yes
State FE	Yes	Yes	Yes	Yes	Yes	Yes	Yes	Yes

Notes: The sample used in this analysis excludes five early expansion states (CA, MA, MN, WA, DC), as well as states that expanded after 2015 but before 2020 (AK, IN, LA, ME, MT, VA). Columns (1) to (4) are estimated coefficients based on full samples. Columns (5) to (8) are estimated coefficients based on subsample with annual income above 100% FPL. Regressions control for respondents' age, sex, race, level of education, household income, citizenship status, marriage status, and employment status. The standard errors clustered at CPUMA level are in the parentheses. $***$, $**$ and $*$ indicate significance at levels 1%, 5%, and 10%.

Table E8: Impact of Housing-Related Expenses on Health Insurance Coverage by Rent Burden and Income Level

	All Income Level				Income Above 100 FPL			
	Uninsured	Medicaid	Employer	Direct	Uninsured	Medicaid	Employer	Direct
	(1)	(2)	(3)	(4)	(5)	(6)	(7)	(8)
$HighRent \times Expanded \times Post$	0.002	0.014***	−0.008	−0.012***	0.006	0.013**	−0.008	−0.017***
	(0.006)	(0.006)	(0.005)	(0.003)	(0.006)	(0.006)	(0.007)	(0.004)
$Expanded \times Post$	−0.039***	0.082***	−0.028***	−0.016***	−0.035***	0.079***	−0.029***	−0.017***
	(0.004)	(0.005)	(0.004)	(0.003)	(0.004)	(0.004)	(0.004)	(0.003)
No. of Observations	999,435	999,435	999,435	999,435	773,283	773,283	773,283	773,283
Year FE	Yes	Yes	Yes	Yes	Yes	Yes	Yes	Yes
State FE	Yes	Yes	Yes	Yes	Yes	Yes	Yes	Yes

Notes: The sample used in this analysis excludes five early expansion states (CA, MA, MN, WA, DC), as well as states that expanded after 2015 but before 2020 (AK, IN, LA, ME, MT, VA). Columns (1) to (4) are estimated coefficients based on full samples. Columns (5) to (8) are estimated coefficients based on subsample with annual income above 100% FPL. Regressions control for respondents' age, sex, race, level of education, household income, citizenship status, marriage status, and employment status. The standard errors clustered at CPUMA level are in the parentheses. $***$, $**$ and $*$ indicate significance at levels 1%, 5%, and 10%.

E.5 Alternative Measurements

Table E9: Impact of Medicaid Expansion on Health Insurance Coverage: Use Overcrowding Dummy Instead of High Rent Dummy

	All Income Level				Income Above 100 FPL			
	Uninsured	Medicaid	Employer	Direct	Uninsured	Medicaid	Employer	Direct
	(1)	(2)	(3)	(4)	(5)	(6)	(7)	(8)
Overcrowding × *Expanded* × *Post*	0.008*	0.011***	−0.016***	−0.003	0.003	0.013***	−0.010*	−0.005*
	(0.004)	(0.004)	(0.005)	(0.003)	(0.005)	(0.004)	(0.005)	(0.003)
Expanded × *Post*	−0.037***	0.077***	−0.022***	−0.017***	−0.031***	0.069***	−0.023***	−0.016***
	(0.003)	(0.004)	(0.004)	(0.002)	(0.003)	(0.003)	(0.004)	(0.003)
No. of Observations	1,969,099	1,969,099	1,969,099	1,969,099	1,490,705	1,490,705	1,490,705	1,490,705
Year FE	Yes	Yes	Yes	Yes	Yes	Yes	Yes	Yes
State FE	Yes	Yes	Yes	Yes	Yes	Yes	Yes	Yes

Notes: The sample used in this analysis excludes five early expansion states (CA, MA, MN, WA, DC), as well as states that expanded after 2015 but before 2020 (AK, IN, LA, ME, MT, VA). Columns (1) to (4) are estimated coefficients based on full samples. Columns (5) to (8) are estimated coefficients based on subsample with annual income above 100% FPL. Regressions control for respondents' age, sex, race, level of education, household income, citizenship status, marriage status, and employment status. The standard errors clustered at CPUMA level are in the parentheses. ∗∗∗, ∗∗ and ∗ indicate significance at levels 1%, 5%, and 10%.

Table E10: Impact of Medicaid Expansion on Health Insurance Coverage: Use High Mortgage Dummy Instead of High Rent Dummy

	All Income Level				Income Above 100% FPL			
	Uninsured	Medicaid	Employer	Direct	Uninsured	Medicaid	Employer	Direct
	(1)	(2)	(3)	(4)	(5)	(6)	(7)	(8)
HighMortgage × *Expanded* × *Post*	0.002	0.016***	−0.005	−0.014***	0.007**	0.009***	−0.002	−0.014***
	(0.003)	(0.003)	(0.003)	(0.003)	(0.003)	(0.003)	(0.004)	(0.003)
Expanded × *Post*	−0.024***	0.059***	−0.016***	−0.016***	−0.019***	0.051***	−0.015***	−0.015***
	(0.003)	(0.003)	(0.003)	(0.002)	(0.003)	(0.003)	(0.003)	(0.002)
No. of Observations	3,123,540	3,123,540	3,123,540	3,123,540	2,803,259	2,803,259	2,803,259	2,803,259
Year FE	Yes	Yes	Yes	Yes	Yes	Yes	Yes	Yes
State FE	Yes	Yes	Yes	Yes	Yes	Yes	Yes	Yes

Notes: The sample used in this analysis exclude any states that implement Medicaid expansion before 2014 or later than 2014. That is, we exclude 5 early expansion states: CA, DC, MA, MN, WA; and 6 late expansion states. Columns (1) to (4) are estimated coefficients based on full samples. Columns (5) to (8) are estimated coefficients based on subsample with annual income above 100 federal poverty line. Regressions control for respondents' age, sex, race, level of education, income-to-poverty ratio, citizenship status, marriage status, and employment status. The standard errors clustered at CPUMA level are in the parentheses. ∗∗∗, ∗∗ and ∗ indicate significance at levels 1%, 5%, and 10%.

E.6 Other Robustness Check

Table E11: Standard errors clustered at state level

	All Income Level				Income Above 100 FPL			
	Uninsured	Medicaid	Employer	Direct	Uninsured	Medicaid	Employer	Direct
	(1)	(2)	(3)	(4)	(5)	(6)	(7)	(8)
HighRent × *Expanded* × *Post*	0.002	0.015^{**}	-0.008	-0.010^{***}	0.006	0.015^{**}	-0.007	-0.015^{**}
	(0.006)	(0.007)	(0.006)	(0.003)	(0.006)	(0.006)	(0.006)	(0.004)
Expanded × *Post*	-0.037^{***}	0.075^{***}	-0.024^{***}	-0.014^{**}	-0.033^{***}	0.071^{***}	-0.025^{***}	-0.013^{**}
	(0.008)	(0.007)	(0.006)	(0.005)	(0.007)	(0.006)	(0.007)	(0.006)
No. of Observations	1,969,099	1,969,099	1,969,099	1,969,099	1,490,705	1,490,705	1,490,705	1,490,705
Year FE	Yes	Yes	Yes	Yes	Yes	Yes	Yes	Yes
State FE	Yes	Yes	Yes	Yes	Yes	Yes	Yes	Yes

Notes: The sample used in this analysis excludes five early expansion states (CA, MA, MN, WA, DC), as well as states that expanded after 2015 but before 2020 (AK, IN, LA, ME, MT, VA). Columns (1) to (4) are estimated coefficients based on full samples. Columns (5) to (8) are estimated coefficients based on subsample with annual income above 100% FPL. Regressions control for respondents' age, sex, race, level of education, household income, citizenship status, marriage status, and employment status. The standard errors clustered at state level are in the parentheses. $***$, $**$ and $*$ indicate significance at levels 1%, 5%, and 10%.

Table E12: Robustness Check: Excluding the Year 2014 to Address Transition Effects of Medicaid Expansion

	All Income Level				Income Above 100 FPL			
	Uninsured	Medicaid	Employer	Direct	Uninsured	Medicaid	Employer	Direct
	(1)	(2)	(3)	(4)	(5)	(6)	(7)	(8)
HighRent × *Expanded* × *Post*	0.002	0.015^{***}	-0.008^{*}	-0.010^{***}	0.006	0.015^{***}	-0.008	-0.015^{***}
	(0.004)	(0.004)	(0.004)	(0.003)	(0.004)	(0.004)	(0.005)	(0.003)
Expanded × *Post*	-0.037^{***}	0.075^{***}	-0.024^{***}	-0.014^{***}	-0.033^{***}	0.071^{***}	-0.025^{***}	-0.013^{***}
	(0.003)	(0.003)	(0.003)	(0.002)	(0.003)	(0.003)	(0.003)	(0.002)
No. of Observations	1,744,023	1,744,023	1,744,023	1,744,023	1,321,608	1,321,608	1,321,608	1,321,608
Year FE	Yes	Yes	Yes	Yes	Yes	Yes	Yes	Yes
State FE	Yes	Yes	Yes	Yes	Yes	Yes	Yes	Yes

Notes: The sample used in this analysis excludes five early expansion states (CA, MA, MN, WA, DC), as well as states that expanded after 2015 but before 2020 (AK, IN, LA, ME, MT, VA). Columns (1) to (4) are estimated coefficients based on full samples. Columns (5) to (8) are estimated coefficients based on subsample with annual income above 100% FPL. Regressions control for respondents' age, sex, race, level of education, household income, citizenship status, marriage status, and employment status. The standard errors clustered at state level are in the parentheses. $***$, $**$ and $*$ indicate significance at levels 1%, 5%, and 10%.

Table E13: Robustness Check: Including Years 2008 and 2009 in the Sample

	All Income Level				Income Above 100 FPL			
	Uninsured	Medicaid	Employer	Direct	Uninsured	Medicaid	Employer	Direct
	(1)	(2)	(3)	(4)	(5)	(6)	(7)	(8)
HighRent × *Expanded* × *Post*	−0.001	0.018^{***}	-0.007^{*}	-0.009^{***}	0.004	0.017^{***}	−0.007	-0.014^{***}
	(0.004)	(0.004)	(0.004)	(0.002)	(0.004)	(0.004)	(0.005)	(0.003)
Expanded × *Post*	-0.034^{***}	0.077^{***}	-0.026^{***}	-0.017^{***}	-0.029^{***}	0.073^{***}	-0.028^{***}	-0.016^{***}
	(0.003)	(0.003)	(0.003)	(0.002)	(0.003)	(0.003)	(0.003)	(0.002)
No. of Observations	2,348,576	2,348,576	2,348,576	2,348,576	1,780,643	1,780,643	1,780,643	1,780,643
Year FE	Yes	Yes	Yes	Yes	Yes	Yes	Yes	Yes
State FE	Yes	Yes	Yes	Yes	Yes	Yes	Yes	Yes

Notes: The sample used in this analysis excludes five early expansion states (CA, MA, MN, WA, DC), as well as states that expanded after 2015 but before 2020 (AK, IN, LA, ME, MT, VA). Columns (1) to (4) are estimated coefficients based on full samples. Columns (5) to (8) are estimated coefficients based on subsample with annual income above 100% FPL. Regressions control for respondents' age, sex, race, level of education, household income, citizenship status, marriage status, and employment status. The standard errors clustered at state level are in the parentheses. $***$, $**$ and $*$ indicate significance at levels 1%, 5%, and 10%.

Table E14: Robustness Check: Including Early Medicaid Expansion States in the Analysis

	All Income Level				Income Above 100 FPL			
	Uninsured	Medicaid	Employer	Direct	Uninsured	Medicaid	Employer	Direct
	(1)	(2)	(3)	(4)	(5)	(6)	(7)	(8)
HighRent × *Expanded* × *Post*	0.001	0.017^{***}	-0.009^{**}	-0.011^{***}	0.004	0.020^{***}	-0.010^{**}	-0.016^{***}
	(0.004)	(0.003)	(0.004)	(0.002)	(0.004)	(0.004)	(0.005)	(0.003)
Expanded × *Post*	-0.042^{***}	0.084^{***}	-0.026^{***}	-0.013^{***}	-0.040^{***}	0.081^{***}	-0.027^{***}	-0.012^{***}
	(0.004)	(0.003)	(0.003)	(0.002)	(0.004)	(0.003)	(0.003)	(0.002)
No. of Observations	2,483,054	2,483,054	2,483,054	2,483,054	1,901,379	1,901,379	1,901,379	1,901,379
Year FE	Yes	Yes	Yes	Yes	Yes	Yes	Yes	Yes
State FE	Yes	Yes	Yes	Yes	Yes	Yes	Yes	Yes

Notes: The sample used in this analysis excludes states that expanded after 2015 but before 2020 (AK, IN, LA, ME, MT, VA). Columns (1) to (4) are estimated coefficients based on full samples. Columns (5) to (8) are estimated coefficients based on subsample with annual income above 100% FPL. Regressions control for respondents' age, sex, race, level of education, household income, citizenship status, marriage status, and employment status. The standard errors clustered at state level are in the parentheses. $***$, $**$ and $*$ indicate significance at levels 1%, 5%, and 10%.
